# Supplementary material for: Natural-Product-Inspired Microwave-Assisted Synthesis of Novel Spirooxindoles as Antileishmanial Agents: Synthesis, Stereochemical Assignment, Bioevaluation, SAR, and Molecular Docking Studies
Source: Molecules. 2023 Jun 16;28(12):4817. doi: 10.3390/molecules28124817 (PMC10302753; doi:10.3390/molecules28124817)
Supplement: Supplementary file 1 [file molecules-28-04817-s001.zip › molecules-2403296-supplementary.pdf]

Supporting Information

Natural-Product-Inspired Microwave-Assisted Synthesis of Novel Spirooxindoles as Antileishmanial Agents: Synthesis, Stereochemical Assignment, Bioevaluation, SAR, and Molecular Docking Studies

Nawal Kishore Sahu [1,2,†], Ritu Sharma [1,†], Kshirsagar Prasad Suhas [3], Jyoti Joshi [4], Kunal Prakash [5], Richa Sharma [1], Ramendra Pratap [5], Xiwen Hu [6], Sukhbir Kaur [4], Mukesh Jain [1], Carmine Coluccini [7,*], Paolo Coghi [6,*] and Sandeep Chaudhary [1,3,*]

[1] Laboratory of Organic and Medicinal Chemistry (OMC Lab), Department of Chemistry, Malaviya National Institute of Technology, Jawaharlal Nehru Marg, Jaipur 302017, India; nawal.chemistry@gmail.com (N.K.S.); rtsharma312@gmail.com (R.S.); richasharmachem1@gmail.com (R.S.); mjain.chy@mnit.ac.in (M.J.)
[2] Department of Chemistry, Government Engineering College, Bharatpur 321303, India
[3] Department of Medicinal Chemistry, National Institute of Pharmaceutical Education and Research, Raebareli (NIPER-R), New Transit Campus, Bijnor-Sisendi Road, Sarojini Nagar, Near CRPF Base Camp, Lucknow 226002, India; kshirsagarprasad06@gmail.com
[4] Parasitology Laboratory, Department of Zoology, Panjab University, Chandigarh 160014, India; jyotijoshi86@gmail.com (J.J.); puzoology@yahoo.com (S.K.)
[5] Department of Chemistry, University of Delhi, North Campus, Delhi 110007, India; kunp93@gmail.com (K.P.); ramendrapratap@gmail.com (R.P.)
[6] School of Pharmacy, Macau University of Science and Technology, Avenida Wai Long, Taipa, Macau, China; 1909853vpa11003@student.must.edu.mo
[7] Institute of New Drug Development, College of Medicine, China Medical University, No. 91, Hsueh-Shih Road, Taichung 40402, Taiwan
* Correspondence: carmine.coluccini@mail.cmu.edu.tw (C.C.); coghips@must.edu.mo (P.C.); schaudhary.chy@mnit.ac.in or schaudhary.chy@niperraebareli.edu.in (S.C.); Tel.: +91-141-2713319 or +91-522-2975587 (S.C.); Fax: +91-141-2529029 (S.C.).
† These authors contributed equally to this work.

S. No.	Contents	Page No.
1.	General experimental conditions	S3
2.	General Procedure for the Synthesis of Chalcones **21a-f**	S3
3.	General Procedure (GP) for the Synthesis of Spirooxindole Derivatives, **23a-f**, **24a-f,** and **25a-g**.	S3
4.	Biological Methods: Parasite strain and culture conditions, *In-vitro* anti-leishmanial activity: Trypan blue dye exclusion method, and Plasmid relaxation assay	S3-S4
5.	Characterization data of spirooxindole derivatives **(23a-f, 24a-f, 25a-g)**	S4-S10

**6.**	**X-ray Crystallography:** Single crystal data of spirooxindole **23f**	**S11-S18**
**7.**	^{1}H & ^{13}C NMR spectral data of all the synthesized compounds **23a-f**, **24a-f** and **25a-g**.	**S19-S37**

Experimental Details & Characterization Data

General experimental

After oven drying, all glass apparatus was utilised. High-quality reagents such as Sigma Aldrich (USA), TCI Chemicals (Tokyo, Japan), and Spectrochem (India) were used. Prior to usage, laboratory-grade commercial reagents and solvents were purified using conventional techniques. The IR spectra were recorded on a Bruker ECO-ATR spectrometer in cm^{-1}. The Jeol resonance spectrometer (400 MHz for ^{1}H; 100 MHz for ^{13}C) was used to record NMR spectra. $CDCl_3$ and DMSO-*d6* are used as the residual solvents. Chemical shifts (δ) from low to high fields are given in parts per million (ppm). A Gevo G-2 S Q-TOF was used to perform electrospray emission mass spectrometry (ESI-MS) and high-resolution mass spectrometry (HRMS). Melting points (mp, °C) in an open capillary were determined using a manual melting point apparatus. Analytical thin-layer chromatography was carried out using Merck TLC Silica Gel F_{254} plates under UV light (λ = 254 nm). Rankem silica gel (particle size: 100-200 mesh) obtained from Rankem™ (India) was used for column chromatography. The microwave-assisted synthesis was carried out in a microwave synthesis reactor (Monowave 200, manufactured by Anton Paar) equipped with continuous stirring and infrared temperature sensors. The Optical rotation of chiral compounds was taken in

Polarimeter (MCP 100, manufactured by Anton Paar) at 20 °C. The docking studies of compounds were carried out using Discovery Studio visualizer software.

General Procedure for the Synthesis of Chalcones 21a-f

The synthesis of target compounds begins with the production of substituted chalcone. Methanol (15 mL) was used to dissolve the substituted acetophenones (7.1 mmol), substituted aldehydes (7.1 mmol), and sodium hydroxide (17.78 mmol), which were then agitated for 7 hours at room temperature. After stirring, the reaction mixture was then added to the crushed ice. In order to produce chalcones **21a-f,** the precipitate of the crude product was filtered under vacuum, washed with distilled water, air-dried, and recrystallized with ethanol.

General Procedure (GP) for the Synthesis of Spirooxindole Derivatives, 23a-f, **24a-f,** and **25a-g**.

A mixture of chalcone (0.38 mmol, 1 equiv.), isatin (0.57 mmol, 1.5 equiv.), amino acid (0.57 mmol, 1.5 equiv.), and carbinol (CH_3OH, 10 mL) was added in a microwave vial (10 mL). The reaction mixture was microwave-irradiated in a microwave synthesis reactor (Anton Paar, Monowave 200) with the following conditions: temperature 80°C, rpm: 600, and time: 5 min. The reaction was evaluated using TLC in an ethyl acetate: hexane (3:7) solvent system. When the reaction was complete, the crude product was extracted with ethyl acetate. After the solvent was removed under high vacuum, the pure product was obtained by column chromatography on silica gel using eluting solvents of ethyl acetate and hexane (7:3 v/v) as the mobile phase. The reaction yields range from 71 to 98%.

Biological Methods

Parasite strain and culture conditions:

The SSG-sensitive Indian strain of *Leishmania donovani* (MHOM/IN/1983/AG83) obtained from CSIR-IICB, Kolkata, was used for the study. The promastigotes of these strains were maintained in Medium 199+10% FCS (Foetal Calf Serum) by serial sub-culturing at an ambient temperature of 22±1°C every 72 hours.

***In vitro* antileishmanial activity**

Trypan blue dye exclusion method

For carrying out the antileishmanial activity *in vitro*, promastigotes of *Leishmania donovani* were used. The promastigotes were harvested from the culture vials, counted, and 2×10^6 cells/well were seeded in a 48-well culture plate. Further, compounds at various concentrations (2 μg/mL, 4 μg/mL, 8 μg/mL and 16 μg/mL) were added in triplets. No drug was added to the well serving as a blank, and amphotericin B was added to the well serving as the positive control. The plate was incubated at 22 ± 1°C in the BOD incubator for 72 hours. After 72 hours, each well was counted for the number of viable parasites using the trypan blue dye exclusion method, and the percentage growth inhibition was calculated by using the formula:

$$\text{Percentage viability} = \frac{\text{No. of viable cells in treated well}}{\text{No. of viable cells in blank well}} \times 100$$

Percentage growth inhibition = 100 – percentage viability

IC_{50} (inhibitory concentration at which 50% of the parasites were dead) was obtained by plotting a linear dose-response curve in SPSS software (Version 23).

Plasmid relaxation assay

The relaxation of supercoiled plasmid DNA is the method used for the determination of LTopIB activity. Various doses of each compound were treated with one unit of pure LTopIB (the enzyme to relax 0.5 μg of supercoiled DNA for 30 min at 37 °C) for 20 min at 4 °C. The reaction mixture including 0.5 μg of supercoiled *p*Bluescript SK(−) plasmid, 10 mM Tris-HCl buffer pH 7.5, 5 mM $MgCl_2$, 0.1 mM EDTA, 15 μg/mL bovine serum albumin, and 150 mM KCl was then added in a final volume of 20 μL. After 30 minutes at 37 °C, the reaction mixtures were stopped by adding 4 µL of loading buffer, which included 5% sarkosyl, 0.12% bromophenol blue, and 25% glycerol. By electrophoresis, the topoisomers were separated in 1% agarose gels were electrophoresed at 2 V/cm for 16 hours in a 0.1 M Tris-borate-EDTA buffer (pH 8.0) after being stained with ethidium bromide (0.5 μg/mL). Plotting the percentage of supercoiled DNA versus drug concentrations allowed researchers to determine the 50% inhibition concentration (IC_{50}) values of LTopIB inhibition as the 50% reduction of supercoiled DNA.

Characterization data of spirooxindole derivatives (23a-f, 24a-f, 25a-g).

(1'*S*,2'*R*,3*S*,7a'*S*)-1'-(4-chlorophenyl)-2'-(4-fluorobenzoyl)-5-methyl-1',2',5',6',7',7a'-hexahydrospiro [indoline-3,3'-pyrrolizin]-2-one (23a): The product was synthesized utilizing the microwave-irradiated approach, and purified by column chromatography over silica gel using hexane/EtOAc (8:2) as an eluent; Physical Appearance: light yellow solid; Yield: 98%; R_f (EtOAc/hexane: 1:1) = 0.48; m.p. 182-184 ºC; $[\alpha]^{20}$ = -6.34º; FT-IR (KBr, ν max/cm^{-1}) 3736.66, 3614.43, 2924.65, 2862.34, 2357.42, 1703.18, 1597.66, 1489.73, 1226.66, 1090.56, 947.62, 817.90; ^{1}H NMR (400 MHz, $CDCl_3$) δ 7.70 (s, 1H), 7.45 – 7.39 (m, 4H), 7.28 – 7.26 (m, 2H), 6.98 (s, 1H), 6.91 – 6.81 (m, 3H), 6.43 (d, *J* = 8 Hz, 1H), 4.78 (d, *J* = 11.2 Hz, 1H), 4.21 – 4.15 (m, 1H), 3.87 – 3.81 (m, 1H), 2.70 – 2.56 (m, 2H), 2.32 (s, 3H), 2.04 – 1.87 (m, 4H); ^{13}C NMR (100 MHz, $CDCl_3$) δ 195.45, 180.71, 138.32, 137.96, 133.52, 133.49, 132.84, 131.98, 130.65, 130.56, 130.00, 129.52, 128.93, 128.14, 124.90, 115.46, 115.25, 109.78, 73.61, 71.92, 64.69, 52.33, 48.24, 36.71, 30.64, 29.77, 27.31, 21.29; HRMS (ESI) calcd. for $C_{28}H_{24}ClFN_2O_2$ $[M+H]^+$: 475.1583; found 475.1587.

(1'*S*,2'*R*,3*S*,7a'*S*)-5-fluoro-2'-(4-fluorobenzoyl)-1'-(4-methoxyphenyl)-1',2',5',6',7',7a'-hexahydro spiro[indoline-3,3'-pyrrolizin]-2-one (23b): The product was synthesized utilizing the microwave-irradiated approach, and purified by column chromatography over silica gel using hexane/EtOAc (7:3) as an eluent; Physical Appearance: light yellow solid; Yield: 66%; R_f (EtOAc/hexane: 1:1) = 0.45; m.p. 161-163 ºC; $[\alpha]^{20}$ = -9.34º; FT-IR (KBr, ν max/cm^{-1}) 3736.63, 2923.44, 2857.29, 2357.28, 1708.92, 1596.95, 1468.94, 1300.00, 1237.16, 1181.69, 1025.49, 813.43, 771.52; ^{1}H NMR (400 MHz, $CDCl_3$) δ 8.65 (s, 1H), 7.50 – 7.47 (m, 2H), 7.40 – 7.37 (m, 2H), 7.02 – 6.99 (m, 1H), 6.87 – 6.81(m, 5H), 6.59 – 6.55 (m, 1H), 4.82 (d, *J* = 11.6 Hz, 1H), 4.21 – 4.15 (m, 1H), 3.83 – 3.77 (m, 1H), 3.74 (s, 3H), 2.65 – 2.61 (m, 2H), 2.04 – 1.98 (m, 1H), 1.94 – 1.88 (m, 2H), 1.73 – 1.67 (m, 1H);^{13}C NMR (100 MHz, $CDCl_3$) δ 195.11, 181.38,

166.97, 164.43, 159.95, 158.73, 157.55, 136.52, 133.43, 133.40, 131.28, 130.75, 130.66, 129.04, 126.94, 126.87, 116.28, 116.04, 115.54, 115.33, 115.28, 114.25, 110.84, 110.76, 74.15, 72.03, 64.48, 55.29, 52.29, 48.15, 30.83, 27.55;HRMS (ESI) calcd. for $C_{28}H_{24}F_2N_2O_3$ $[M+H]^+$: 475.1828; found 475.1835.

(1'*S*,2'*R*,3*S*,7a'*S*)-2'-(4-bromobenzoyl)-5-fluoro-1'-(4-methoxyphenyl)-1',2',5',6',7',7a'-hexahydro spiro[indoline-3,3'-pyrrolizin]-2-one (23c): The product was synthesized utilizing the microwave-irradiated approach, and purified by column chromatography over silica gel using hexane/EtOAc (7:3) as an eluent; Physical Appearance: off white solid; Yield: 62%; R_f (EtOAc/hexane: 1:1) = 0.46; m.p. 126-128 ºC; $[\alpha]^{20}$ = -8.33º; FT-IR (KBr, ν max/cm^{-1}) 3737.67, 3315.31, 2920.61, 2854.37, 2357.91, 1728.24, 1473.76, 1391.61, 1257.77, 1176.07, 1032.00, 981.91, 854.02, 810.50, 768.51, 678.70; ^{1}H NMR (400 MHz, $CDCl_3$) δ 7.65 (s, 1H), 7.39 – 7.29 (m, 6H), 6.99 – 6.97 (m, 1H), 6.85 – 6.82 (m, 3H), 6.53 – 6.50 (m, 1H), 4.81 (d, J = 11.6 Hz, 1H), 4.21 – 4.16 (m, 1H), 3.80 – 3.77 (m, 1H), 3.75 (s, 3H), 2.64 – 2.61 (m, 2H), 2.06 – 1.87 (m, 4H); ^{13}C NMR (100 MHz, $CDCl_3$) δ 195.82, 180.62, 159.95, 158.77, 157.54, 136.29, 136.26, 135.75, 131.63, 131.17, 129.54, 129.04, 128.39, 126.85, 126.77, 116.32, 116.09, 115.58, 115.33, 114.26, 110.66, 110.58, 73.88, 72.00, 64.59, 55.31, 52.29, 48.14, 30.78, 27.54; HRMS (ESI) calcd. for $C_{28}H_{24}F_2N_2O_3$ $[M+H]^+$: 535.1027; found 535.1037 .

(1'*S*,2'*R*,3*S*,7a'*S*)-2'-(4-bromobenzoyl)-5-methoxy-1'-(4-methoxyphenyl)-1',2',5',6',7',7a'-hexahydro spiro[indoline-3,3'-pyrrolizin]-2-one (23d): The product was synthesized utilizing the microwave-irradiated approach, and purified by column chromatography over silica gel using hexane/EtOAc (7:3) as an eluent; Physical Appearance: Pale yellow solid; Yield: 93%; R_f (EtOAc/hexane: 1:1) = 0.46; m.p. 107-109 ºC; $[\alpha]^{20}$ = +9.13º; FT-IR (KBr, ν max/cm^{-1}) 2926.14, 2856.60, 1710.37, 1585.99, 1479.19, 1392.20, 1191.17, 1031.76, 807.41, 641.56; ^{1}H NMR (400 MHz, $CDCl_3$) δ 7.56 (m, 1H), 7.41 (d, *J* = 8.8 Hz, 2H), 7.34 – 7.29 (m, 4H), 6.86 – 6.83 (m, 3H), 6.68 - 6.66 (m, 1H), 6.51 – 6.48 (m, 1H), 4.81 (d, J = 11.2 Hz, 1H), 4.23 – 4.17 (m, 1H), 3.81 (s, 3H), 3.76 (s, 3H), 2.71 – 2.60 (m, 2H), 2.06 – 1.98 (m, 1H), 1.95 – 1.86 (m, 2H), 1.75 – 1.68 (m, 2H); ^{13}C NMR (100 MHz, $CDCl_3$) δ 196.00, 180.57, 158.68, 155.52, 135.92, 133.76, 131.52, 129.55, 129.10, 128.14, 126.39, 114.91, 114.20, 113.94, 110.33, 74.00, 72.04, 64.65, 56.00, 55.30, 52.22, 48.20, 30.83, 27.50; HRMS (ESI) calcd. for $C_{29}H_{27}BrN_2O_4$ $[M+H]^+$: 547.1227; found 547.1217.

(1'*S*,2'*R*,3*S*,7a'*S*)-2'-(4-bromobenzoyl)-1'-(4-methoxyphenyl)-5-nitro-1',2',5',6',7',7a'-hexahydrospiro [indoline-3,3'-pyrrolizin]-2-one (23e): The product was synthesized utilizing the microwave-irradiated approach, and purified by column chromatography over silica gel using hexane/EtOAc (7:3) as an eluent; Physical Appearance: light brown solid; Yield: 83%; R_f (EtOAc/hexane: 1:1) = 0.46; m.p. 111-113 ºC; $[\alpha]^{20}$ = +8.45º; FT-IR (KBr, ν max/cm^{-1}) 3737.96, 3676.06, 2921.84, 2357.32, 1736.40, 1682.32, 1512.82, 1335.88, 1247.44, 1027.44, 1024.09, 825.82, 746.30; ^{1}H NMR (400 MHz, $CDCl_3$) δ 8.13 – 8.11 (m, 2H), 7.81 (s, 1H), 7.39 – 7.30 (m, 6H), 6.85 – 6.83 (m, 2H), 6.72 – 6.69 (m, 1H), 4.86 (d, *J* = 11.2 Hz, 1H), 4.24 – 4.19 (m, 1H), 3.89 – 3.84 (m, 1H), 3.75 (s, 3H), 2.63 – 2.59 (m, 2H), 2.07 – 1.92 (m, 3H), 1.79 – 1.72 (m, 1H); ^{13}C NMR (100 MHz, $CDCl_3$) δ 195.32, 180.45, 158.91, 145.87, 143.37, 143.16, 135.51, 131.89,

130.56, 129.48, 129.00, 128.84, 126.51, 126.25, 123.48, 73.11, 71.97, 64.55, 55.31, 52.49, 48.18, 30.60, 27.63; HRMS (ESI) calcd. for $C_{28}H_{24}BrN_3O_5$ $[M+H]^+$: 562.0972; found 562.1021.

(1'*S*,2'*R*,3*S*,7a'*S*)-2'-benzoyl-5-bromo-1'-phenyl-1',2',5',6',7',7a'-hexahydrospiro[indoline-3,3'-pyrrolizin]-2-one (23f) : The product was synthesized utilizing the microwave-irradiated approach, and purified by column chromatography over silica gel using hexane/EtOAc (7:3) as an eluent; Physical Appearance: creamy white solid; Yield: 71%; R_f (EtOAc/hexane = 2:3): 0.37; m.p.: 175-177 ºC; $[\alpha]^{20}$ = +12.61º; FT-IR (KBr, ν max/cm^{-1}) 3437.04, 1740.57, 1713.85, 1674.72, 1469.14, 737.54, 692.44; ^1H NMR (400 MHz, $CDCl_3$) δ 8.53 (s, 1H), 7.49 (d, *J* = 7.6 Hz, 2H), 7.1 (d, *J* = 7.6 Hz, 2H), 7.35 – 7.28 (m, 4H), 7.26 – 7.23 (m, 1H), 7.21-7.13 (m, 3H), 6.48 (d, *J* = 8.4 Hz, 1H), 4.92 (d, *J* = 11.6 Hz, 1H), 4.24 – 4.19 (m, 1H), 3.90 – 3.83 (m, 1H), 2.69 – 2.58 (m, 2H), 2.04 – 1.73 (m, 4H); ^{13}C NMR (100 MHz, $CDCl_3$) δ 196.72, 180.88, 139.73, 139.51, 137.03, 133.15, 132.46, 130.49, 128.81, 128.30, 128.16, 127.98, 127.48, 127.16, 115.19, 111.63, 73.75, 72.18, 64.59, 52.93, 48.28, 30.76, 27.52; HRMS (ESI) calcd for $C_{27}H_{24}BrN_2O_2$($M+H^+$): 487.1016; found, 487.1012.

(3*S*,3'*R*,4'*S*,5'*S*)-5'-benzyl-4'-(4-chlorophenyl)-5-methoxy-3'-(4-nitrobenzoyl)spiro[indoline-3,2'-pyrrolidin]-2-one (24a): The product was synthesized utilizing the microwave-irradiated approach, and purified by column chromatography over silica gel using hexane/EtOAc (7:3) as an eluent; Physical Appearance: off white solid; Yield: 89%; R_f (EtOAc/hexane: 1:1) = 0.47; m.p. 188-190 ºC; $[\alpha]^{20}$ = -9.36º; FT-IR (KBr, ν max/cm^{-1}) 3741.44, 3406.50, 2925.60, 2856.39, 2359.93, 1707.29, 1525.89, 1341.38, 1196.99, 968.10, 810.00, 738.94, 694.52; ^1H NMR (400 MHz, $CDCl_3$) δ 7.99 – 7.97 (d, J = 8.4 Hz, 2H), 7.53 (s, 1H), 7.48 – 7.43 (m, 3H), 7.30 – 7.26 (m, 4H), 7.21– 7.12 (m, 5H), 6.52 – 6.49 (m, 1H), 6.38 (d, *J* = 2 Hz, 1H), 6.26 (d, *J* = 8.8 Hz, 1H), 4.50 (d, *J* = 10.8 Hz, 1H), 4.29 – 4.24 (m, 1H), 3.82 – 3.77 (m, 1H), 3.68 (s, 3H), 2.98 – 2.94 (m, 1H), 2.72 – 2.67 (m, 1H); ^{13}C NMR (100 MHz, $CDCl_3$) δ 195.79, 181.25, 156.39, 150.16, 149.97, 141.45, 141.29, 137.60, 134.80, 132.80, 130.29, 130.06, 129.55, 128.75, 128.64, 128.58, 127.70, 126.88, 126.71, 123.36, 115.14, 111.93, 110.03, 68.68, 64.63, 64.10, 55.98, 52.03, 38.80; HRMS (ESI) calcd. for $C_{32}H_{26}ClN_3O_5$ $[M+H]^+$: 568.1634; found 568.1692.

(3*S*,3'*R*,4'*S*,5'*S*)-5'-benzyl-4'-(4-bromophenyl)-3'-(4-methylbenzoyl)-5-nitrospiro[indoline-3,2'-pyrrolidin]-2-one (24b): The product was synthesized utilizing the microwave-irradiated approach, and purified by column chromatography over silica gel using hexane/EtOAc (7:3) as an eluent; Physical Appearance: off white solid; Yield: 95%; R_f (EtOAc/hexane: 1:1) = 0.42 ; m.p. 121-122 ºC; $[\alpha]^{20}$ = -13.12º; FT-IR (KBr, ν max/cm^{-1}) 3736.91, 2923.49, 2358.09, 1739.91, 1602.85, 1516.32, 1330.77, 1179.63, 1092.61, 751.40, 696.21; ^1H NMR (400 MHz, $CDCl_3$) δ 7.97 – 7.93 (m, 2H), 7.83 (d, *J* = 2.4 Hz, 1H), 7.47 – 7.39 (m, 4H), 7.27 – 7.14 (m, 8H), 6.95 (d, *J* = 8.0 Hz, 2H), 6.51 (d, *J* = 8.4 Hz, 1H), 4.51 (d, *J* = 10.4 Hz, 1H), 4.25 – 4.19 (m, 1H), 3.94 – 3.89 (m, 1H), 2.97 – 2.93 (m, 1H), 2.72 – 2.67 (m, 1H), 2.23 (s, 3H); ^{13}C NMR (100 MHz, $CDCl_3$) δ 195.68, 181.94, 145.37, 144.51, 143.81, 138.09, 137.94, 134.30, 132.12, 130.88, 130.33, 129.30, 129.26, 128.66, 127.81, 126.78, 126.06, 122.27, 121.32, 109.21, 68.27,

65.02, 62.91, 52.24, 39.39, 21.65;HRMS (ESI) calcd. for $C_{32}H_{26}BrN_3O_4$ $[M+H]^+$: 596.118; found 596.1233.

(3S,3'R,4'S,5'S)-5'-benzyl-4'-(4-chlorophenyl)-5-methyl-3'-(4-nitrobenzoyl)spiro[indoline-3,2'-pyrrolidin]-2-one (24c): The product was synthesized utilizing the microwave-irradiated approach, and purified by column chromatography over silica gel using hexane/EtOAc (7:3) as an eluent; Physical Appearance: dark yellow solid; Yield: 88%; R_f (EtOAc/hexane: 1:1) = 0.47 ; m.p. 135-137 ºC; $[\alpha]^{20}$ = -14.12º; FT-IR (KBr, ν max/cm^{-1}) 3742.96, 3618.41, 2919.80, 2852.32, 2361.15, 1695.04, 1522.46, 1341.81, 1088.83, 968.72, 817.89, 691.96; ^{1}H NMR (400 MHz, $CDCl_3$) δ 7.97 (d, *J* = 8.4 Hz, 2H), 7.54 (s, 1H), 7.45 – 7.42 (m, 3H), 7.31 – 7.15 (m, 5H), 7.24 – 7.14 (m, 4H), 6.76 (d, *J* = 6.8 Hz, 1H), 6.52 (s, 1H), 6.26 – 6.22 (m, 1H), 4.50 (d, *J* = 10.4 Hz, 1H), 4.27 – 4.22 (m, 1H), 3.84 – 3.78 (m, 1H), 2.98 – 2.94 (m, 1H), 2.74 – 2.68 (m, 1H), 2.19 (s, 3H); ^{13}C NMR (100 MHz, $CDCl_3$) δ 195.97, 181.21, 149.91, 141.57, 141.33, 137.48, 137.13, 134.79, 133.09, 130.28, 129.97, 129.62, 128.76, 128.62, 128.59, 127.68, 126.87, 126.72, 126.30, 123.32, 109.20, 68.47, 64.70, 64.15, 52.06, 38.66, 21.01; HRMS (ESI) calcd. for $C_{32}H_{26}ClN_3O_4$ $[M+H]^+$: 552.1685; found 552.1700.

(3*S*,3'*R*,4'*S*,5'*S*)-5'-benzyl-4'-(4-chlorophenyl)-3'-(4-fluorobenzoyl)spiro[indoline-3,2'-pyrrolidin]-2-one (24d): The product was synthesized utilizing the microwave-irradiated approach, and purified by column chromatography over silica gel using hexane/EtOAc (7:3) as an eluent; Physical Appearance: light orange solid; Yield: 97%; R_f (EtOAc/hexane: 1:1) = 0.47; m.p. 104-106 ºC; $[\alpha]^{20}$ = +14.10º; FT-IR (KBr, ν max/cm^{-1}) 3738.13, 3223.12, 2922.72, 2358.43, 1708.79, 1476.71, 1238.52, 984.20, 744.78, 690.39; ^{1}H NMR (400 MHz, $CDCl_3$) δ 7.49 – 7.30 (m, 7H), 7.23 – 7.13 (m, 6H), 6.99 – 6.95 (m, 1H), 6.87 – 6.80 (m, 4H), 6.41 (d, *J* = 7.6 Hz, 1H), 4.45 (d, *J* = 10.8 Hz, 1H), 4.24 – 4.19 (m, 1H), 3.85 – 3.80 (m, 1H), 2.97 – 2.93 (m, 1H), 2.71 – 2.66 (m, 1H); ^{13}C NMR (100 MHz, $CDCl_3$) δ 195.49, 181.59, 166.79, 164.25, 139.64, 137.97, 137.71, 133.51, 133.00, 130.26, 129.99, 129.64, 129.21, 129.09, 128.58, 126.63, 125.71, 123.21, 115.47, 115.26, 109.35, 68.63, 64.72, 63.53, 52.08, 38.87; HRMS (ESI) calcd. for $C_{31}H_{24}ClFN_2O_2$ $[M+H]^+$: 511.1583; found 511.1783.

(3*S*,3'*R*,4'*S*,5'*S*)-5'-benzyl-7-bromo-4'-(4-chlorophenyl)-3'-(4-nitrobenzoyl)spiro[indoline-3,2'-pyrrolidin]-2-one (24e): The product was synthesized utilizing the microwave-irradiated approach, and purified by column chromatography over silica gel using hexane/EtOAc (7:3) as an eluent; Physical Appearance: light orange solid; Yield: 83 %; R_f (EtOAc/hexane: 1:1) = 0.46; m.p. 166-168 ºC; $[\alpha]^{20}$ = -8.31º;FT-IR (KBr, ν max/cm^{-1}) 3842.02, 3742.16, 3618.21, 2973.16, 2923.30, 2359.76, 1701.34, 1517.86, 1339.19, 1172.08, 957.65, 743.66, 685.17; ^{1}H NMR (400 MHz, $CDCl_3$) δ 8.01 (d, *J* = 8.8 Hz, 2H), 7.51 (s, 1H), 7.44 – 7.39 (m, 3H), 7.32 – 7.26 (m, 4H), 7.23 – 7.09 (m, 6H), 6.80 – 6.73 (m, 2H), 4.48 (d, *J* = 10.8 Hz, 1H), 4.29 – 4.24 (m, 1H), 3.80 – 3.75 (m, 1H), 2.97 – 2.93 (m, 1H), 2.70 – 2.64 (m, 1H); ^{13}C NMR (100 MHz, $CDCl_3$) δ 195.73, 180.18, 150.07, 141.27, 140.89, 139.05, 137.59, 134.87, 132.08, 130.55, 130.34, 129.46, 128.61, 127.82, 126.81, 124.59, 123.37, 102.71, 69.59, 64.68, 51.77, 39.03; HRMS (ESI) calcd. for $C_{31}H_{23}BrClN_3O_4$ $[M+H]^+$: 616.0633; found 616.0640 .

(3*S*,3'*R*,4'*S*,5'*S*)-5'-benzyl-4'-(4-bromophenyl)-5-iodo-3'-(4-methylbenzoyl)spiro[indoline-3,2'-pyrrolidin]-2-one (24f): The product was synthesized utilizing the microwave-irradiated approach, and purified by column chromatography over silica gel using hexane/EtOAc (7:3) as an eluent; Physical Appearance: light yellow solid; Yield: 98 % ; R_f (EtOAc/hexane: 1:1) = 0.45; m.p. 101-102 ºC; $[\alpha]^{20}$ = -12.11º; FT-IR (KBr, ν max/cm^{-1}) 3843.17, 3738.64, 2922.18, 2359.25, 1704.53, 1464.69, 1177.66, 961.68, 815.11, 689.50; ^{1}H NMR (400 MHz, $CDCl_3$) δ 7.46 – 7.39 (m, 5H), 7.28 – 7.08 (m, 11H), 6.94 (d, J = 8 Hz, 2H), 6.17 (d, J = 7.6 Hz, 1H), 4.46 (d, J = 10.8 Hz, 1H), 4.22 – 4.16 (m, 1H), 3.84 – 3.78 (m, 1H), 2.95 – 2.91 (m, 1H), 2.69 – 2.64 (m, 1H), 2.23 (s, 3H);^{13}C NMR (100 MHz, $CDCl_3$) δ 196.16, 181.09, 144.00, 139.34, 138.40, 137.92, 134.65, 132.04, 130.40, 129.48, 129.05, 128.60, 127.84, 126.82, 121.11, 111.26, 85.46, 64.78, 63.07, 51.95, 38.98, 21.64; HRMS (ESI) calcd. for $C_{32}H_{26}BrIN_2O_2$ $[M+H]^+$: 677.0295; found 677.0294.

(3*S*,3'*R*,4'*S*,5'*S*)-5'-((1*H*-indol-2-yl)methyl)-5-bromo-3'-(4-fluorobenzoyl)-4'-(4-methoxyphenyl)spiro [indoline-3,2'-pyrrolidin]-2-one (25a): The product was synthesized utilizing the microwave-irradiated approach, and purified by column chromatography over silica gel using hexane/EtOAc (7:3) as an eluent; Physical Appearance: light brown solid; Yield: 91%; R_f (EtOAc/hexane: 6:4) = 0.43; m.p. 154-156 ºC; $[\alpha]^{20}$ = +3.22º; FT-IR (KBr, ν max/cm^{-1}) 3740.66, 3362.57, 2924.26, 2852.77, 2357.55, 1706.96, 1602.62, 1510.66, 1462.94, 1241.10, 1178.84, 1095.50, 815.64, 739.36, 638.87; ^{1}H NMR (400 MHz, $CDCl_3$) δ 8.12 (s, 1H), 7.68 (s, 1H), 7.47 – 7.36 (m, 6H), 7.31 (d, J = 8.0 Hz, 1H), 7.15 – 7.12 (m, 1H), 7.05 – 7.02 (m, 3H), 6.89 (d, J = 8.8 Hz, 2H), 6.83 – 6.78 (m, 3H), 6.20 (d, J = 8.4 Hz, 1H), 4.48 (d, J = 10.8 Hz, 1H), 4.28 – 4.23 (m, 1H), 3.88 – 3.83 (m, 1H), 3.78 (s, 3H), 3.14 – 3.10 (m, 1H), 2.84 – 2.78 (m, 1H); ^{13}C NMR (100 MHz, $CDCl_3$) δ 195.55, 181.47, 166.78, 164.25, 158.81, 138.68, 136.24, 133.71, 131.97, 131.83, 131.17, 130.42, 130.32, 129.61, 128.91, 127.88, 122.83, 122.19, 119.59, 118.98, 115.52, 115.30, 114.34, 112.14, 111.34, 110.78, 68.83, 64.75, 63.58, 55.34, 51.95, 28; HRMS (ESI) calcd. for $C_{34}H_{27}BrFN_3O_3$ $[M+H]^+$: 624.1293; found 624.1294.

(3*S*,3'*R*,4'*S*,5'*S*)-5'-((1*H*-indol-2-yl)methyl)-3'-(4-bromobenzoyl)-4'-(4-methoxyphenyl)spiro[indoline-3,2'-pyrrolidin]-2-one (25b): The product was synthesized utilizing the microwave-irradiated approach, and purified by column chromatography over silica gel using hexane/EtOAc (7:3) as an eluent; Physical Appearance: light brown solid; Yield: 75%; R_f (EtOAc/hexane: 6:4) = 0.47; m.p. 118-120 ºC; $[\alpha]^{20}$ = +4.27º; FT-IR (KBr, ν max/cm^{-1}) 3739.37, 3350.15, 2924.66, 2358.94, 1710.54, 1615.64. 1510.92, 1465.00, 1334.73, 1243.36, 1178.13, 1071.73, 998.85, 830.41, 740.30; ^{1}H NMR (400 MHz, $CDCl_3$) δ 8.09 (s, 1H), 7.48 – 7.45 (m, 4H), 7.30 – 7.11 (m, 9H), 6.94 – 6 .88 (m, 3H), 6.81 – 6.75 (m, 2H), 6.36 (d, J = 8 Hz, 1H), 4.49 (d, J = 10.8 Hz, 1H), 4.30 – 4.25 (m, 1H), 3.88 – 3.83 (m, 1H), 3.78 (s, 3H), 3.16 – 3.12 (m, 1H), 2.86 – 2.81 (m, 1H); ^{13}C NMR (100 MHz, $CDCl_3$) δ 196.61, 181.69, 158.74, 139.70, 136.24, 136.02, 131.46, 131.42, 129.61, 129.49, 129.21, 129.15, 127.97, 127.93, 125.63, 123.06, 122.86, 122.11, 119.51,

119.11, 114.29, 112.16, 111.10, 109.36, 68.82, 64.70, 63.84, 55.34, 52.35, 27.99; HRMS (ESI) calcd. for $C_{34}H_{28}BrN_3O_3$ $[M+H]^+$: 606.1387; found 606.1381.

(3*S*,3'*R*,4'*S*,5'*S*)-5'-((1*H*-indol-2-yl)methyl)-5-bromo-4'-(4-chlorophenyl)-3'-(4-fluorobenzoyl)spiro [indoline-3,2'-pyrrolidin]-2-one (25c): The product was synthesized utilizing the microwave-irradiated approach, and purified by column chromatography over silica gel using hexane/EtOAc (7:3) as an eluent; Physical Appearance: dark orange solid; Yield: 71%; R_f (EtOAc/hexane: 1:1) = 0.47; m.p. 138-140 ºC; $[\alpha]^{20}$ = -8.65º; FT-IR (KBr, ν max/cm^{-1}) 3744.85, 2925.84, 2858.05, 2362.33, 1714.67, 1597.61, 1468.52, 1421.12, 1232.15, 1089.95, 1008.23, 814.04, 738.90; ^{1}H NMR (400 MHz, $CDCl_3$) δ 8.09 (s, 1H), 7.57 (s, 1H), 7.48 – 7.24 (m, 9H), 7.17 – 7.13 (m, 1H), 7.06 – 7.03 (m, 3H), 6.84 – 6.80 (m, 3H), 6.23 (d, *J* = 8 Hz, 1H), 4.45 (d, *J* = 10.8 Hz, 1H), 4.31 – 4.26 (m, 1H), 3.87 (t, *J* = 10.8 Hz, 1H), 3.12 – 3.07 (m, 1H), 2.86 – 2.81 (m, 1H); ^{13}C NMR (100 MHz, $CDCl_3$) δ 195.24, 181.27, 138.62, 137.86, 136.24, 133.52, 133.06, 132.11, 131.56, 130.41, 130.31, 130.00, 129.05, 128.92, 127.78, 122.80, 122.28, 119.66, 118.91, 115.64, 115.61, 115.39, 111.90, 111.36, 110.81, 68.77, 64.65, 63.62, 52.00, 28.15; HRMS (ESI) calcd. for $C_{33}H_{24}BrClFN_3O_2$ $[M+H]^+$: 628.0797; found 628.0800.

(3*S*,3'*R*,4'*S*,5'*S*)-5'-((1*H*-indol-2-yl)methyl)-4'-(4-bromophenyl)-3'-(4-cyclohexylbenzoyl)-5-fluoro spiro[indoline-3,2'-pyrrolidin]-2-one (25d): The product was synthesized utilizing the microwave-irradiated approach, and purified by column chromatography over silica gel using hexane/EtOAc (7:3) as an eluent; Physical Appearance: orange brown solid; Yield: 73%;$[\alpha]^{20}$ = -6.72º; R_f (EtOAc/hexane: 6:4) = 0.49; m.p. 112-114 ºC; FT-IR (KBr, ν max/cm^{-1}) 3737.58, 2921.02, 2852.01, 2356.89, 1709.66, 1604.32, 1481.52, 1180.27, 1004.28, 814.57, 737.49; ^{1}H NMR (400 MHz, $CDCl_3$) δ 8.09 (s, 1H), 7.53 – 7.41 (m, 6H), 7.32 – 7.28 (m, 3H), 7.15 – 7.11 (m, 1H), 7.05 – 6.95 (m, 4H), 6.62 – 6.52 (m, 2H), 6.25 – 6.22 (m, 1H), 4.50 (d, *J* = 10.8 Hz, 1H), 4.33 – 4.27 (m, 1H), 3.90 – 3.85 (m, 1H), 3.11 – 3.07 (m, 1H), 2.87 -2.81 (m, 1H), 2.39 – 2.33 (m, 1H), 2.03 – 1.66 (m, 10H); ^{13}C NMR (100 MHz, $CDCl_3$) δ 196.40, 181.87, 160.32, 157.91, 153.88, 138.71, 136.26, 135.62, 134.81, 131.89, 131.29, 131.22, 130.41, 127.95, 127.83, 126.79, 122.78, 122.20, 120.99, 119.57, 118.98, 115.65, 115.41, 113.80, 113.55, 112.03, 111.22, 109.83, 109.75, 69.17, 64.60, 63.36, 52.35, 44.54, 34.03, 29.77, 28.25, 26.66, 26.00, 22.73, 14.20; HRMS (ESI) calcd. for $C_{39}H_{35}BrFN_3O_2$ $[M+H]^+$: 676.1970; found 676.1978.

(3*S*,3'*R*,4'*S*,5'*S*)-5'-((1*H*-indol-2-yl)methyl)-3'-(4-fluorobenzoyl)-4'-(4-methoxyphenyl)-5-methylspiro [indoline-3,2'-pyrrolidin]-2-one (25e): The product was synthesized utilizing the microwave-irradiated approach, and purified by column chromatography over silica gel using hexane/EtOAc (7:3) as an eluent; Physical Appearance: light brown solid; Yield: 87%; R_f (EtOAc/hexane: 1:1) = 0.41; m.p. 103-105 ºC; $[\alpha]^{20}$ = -6.38º; FT-IR (KBr, ν max/cm^{-1}) 3738.02, 3615.96, 2922.43, 2853.52, 2357.16, 1710.90, 1599.16, 1508.54, 1238.76, 1031.57, 816.77, 741.91; ^{1}H NMR (400 MHz, $CDCl_3$) δ 8.06 (s, 1H), 7.48 (d, *J* = 8.8 Hz, 3H), 7.37 – 7.31 (m, 3H), 7.18 – 7.12 (m, 4H), 7.06 – 7.01 (m, 2H), 6.88 (d, J = 8.8 Hz, 2H), 6.81 – 6.77 (m, 2H), 6.70 (d, *J* = 8.4 Hz, 1H), 6.47 (s, 1H), 6.23 (d, *J* = 8 Hz, 1H), 4.51 – 4.48 (d, *J* = 10.8 Hz, 1H), 4.29 – 4.24 (m, 1H), 3.89 – 3.84 (m, 1H), 3.78 (s, 3H), 3.17 – 3.13 (m, 1H), 2.88 – 2.83 (m, 1H), 2.11

(s, 3H).; ^{13}C NMR (100 MHz, $CDCl_3$) δ 195.93, 181.67, 166.66, 158.7 137.09, 136.25, 133.81, 132.61, 131.54, 130.35, 130.26, 129.63, 129.39, 128.03, 126.24, 123.00, 122.12, 119.56, 119.14, 115.32, 115.11, 114.26, 112.11, 111.07, 108.87, 68.98, 64.66, 63.82, 55.33, 52.11, 27.80, 20.96; HRMS (ESI) calcd. for $C_{35}H_{30}FN_3O_3$ $[M+H]^+$: 560.2344; found 560.2345.

(3*S*,3'*R*,4'*S*,5'*S*)-5'-((1*H*-indol-2-yl)methyl)-4'-(4-chlorophenyl)-3'-(4-fluorobenzoyl)-5-nitrospiro [indoline-3,2'-pyrrolidin]-2-one (25f): The product was synthesized utilizing the microwave-irradiated approach, and purified by column chromatography over silica gel using hexane/EtOAc (7:3) as an eluent; Physical Appearance: off white solid; Yield: 72%; R_f (EtOAc/hexane: 1:1) = 0.49; m.p. 172-174 ºC; $[\alpha]^{20}$ = +8.92º; FT-IR (KBr, ν max/cm^{-1}) 3737.36, 3615.92, 2925.29, 2856.15, 2357.07, 1708.55, 1517.21, 1334.45, 1192.41, 1097.14, 964.81, 830.23, 744.18; ^{1}H NMR (400 MHz, DMSO) δ 10.96 (s, 1H), 10.73 (s, 1H), 8.27 (s, 1H), 7.90 – 7.88 (m, 1H), 7.74 (d, *J* = 2 Hz, 1H), 7.47 (d, *J* = 8.8 Hz, 2H), 7.39 – 7.34 (m, 3H), 7.26 – 7.20 (m, 2H), 7.06 – 6.95 (m, 4H), 6.88 – 6.84 (m, 1H), 6.56 (d, *J* = 8.4 Hz, 1H), 4.46 (d, *J* = 10.4 Hz, 1H), 4.07 (s, 1H), 3.84 – 3.79 (m, 1H), 3.65 (d, *J* = 7.2 Hz, 1H), 2.86 – 2.76 (m, 2H);^{13}C NMR (100 MHz, DMSO) δ 196.29, 182.28, 148.66, 142.33, 139.39, 136.62, 133.79, 131.99, 130.84, 130.75, 130.65, 129.13, 127.77, 126.57, 123.85, 121.83, 121.24, 118.64, 118.61, 116.22, 116.00, 111.89, 111.81, 109.74, 79.70, 68.39, 65.33, 63.35, 52.82, 28.80; HRMS (ESI) calcd. for $C_{33}H_{24}ClFN_4O_4$ $[M+H]^+$: 595.1543; found 595.1598.

(3*S*,3'*R*,4'*S*,5'*S*)-5'-((1*H*-indol-2-yl)methyl)-4'-(4-chlorophenyl)-3'-(4-fluorobenzoyl)-5-methoxyspiro [indoline-3,2'-pyrrolidin]-2-one (25g): The product was synthesized utilizing the microwave-irradiated approach, and purified by column chromatography over silica gel using hexane/EtOAc (7:3) as an eluent; Physical Appearance: light brown solid; Yield: 81%; R_f (EtOAc/hexane: 1:1) = 0.43; m.p. 142-145 ºC; $[\alpha]^{20}$ = -7.74º; FT-IR (KBr, ν max/cm^{-1}) 3844.59, 3737.95, 3281.29, 2920.36, 2851.62, 2357.87, 1728.18, 1598.43, 1486.63, 1301.36, 1091.64, 1028.63, 949.87, 813.72, 735.76; ^{1}H NMR (400 MHz, $CDCl_3$) δ 8.10 (s, 1H), 7.53 – 7.46 (m, 4H), 7.39 – 7.36 (m, 2H), 7.31 – 7.29 (m, 3H), 7.16 – 7.01 (m, 4H), 6.82 – 6.78 (m, 2H), 6.48 – 6.45 (m, 1H), 6.34 – 6.27 (m, 2H), 4.46 (d, *J* = 10.8 Hz, 1H), 4.33 – 4.28 (m, 1H), 3.90 – 3.84 (m, 1H), 3.58 (s, 3H), 3.13 – 3.09 (m, 1H), 2.89 – 2.83 (m, 1H); ^{13}C NMR (100 MHz, $CDCl_3$) δ 195.56, 181.85, 166.79, 164.25, 156.12, 138.23, 136.26, 133.55, 133.52, 133.03, 132.91, 130.39, 130.34, 130.25, 130.03, 128.99, 127.89, 123.00, 122.19, 119.61, 119.07, 115.47, 115.25, 114.99, 69.30, 64.56, 63.82, 55.90, 52.25, 22.74; HRMS (ESI) calcd. for $C_{34}H_{27}ClFN_3O_3$ $[M+H]^+$: 580.1798; found 580.1849.

II. X-ray Crystallography

Single crystals of $C_{27}H_{23}BrN_2O_2$ of spirooxindole **23f** were picked up from the mother liquor of DCM which was kept for crystallization using the slow evaporation method at low temperature. A suitable crystal was selected and mounted on Oxford Diffraction diffractometer. The crystal was kept at 293(2) K during data collection. Using Olex2, the structure was solved with the ShelxT structure solution program using Intrinsic Phasing method and refined with the refinement package using minimization.

Crystal Data for $C_{27}H_{23}BrN_2O_2$ (M = 486.09 g/mol): trigonal, space group R-3 (no. 148), a = 22.6902(11) Å, c = 25.8577(11) Å, V = 11529.1(12) Å^3, Z = 107, T = 293(2) K, μ(MoKα) = 1.637 mm^{-1}, $Dcalc$ = 1.419 g/cm^3, 46600 reflections measured (7.814° ≤ 2Θ ≤ 49.99°), 4512 unique (R_{int} = 0.0710, R_{sigma} = 0.0424) which were used in all calculations. The final R_1 was 0.0927 (I > 2σ(I)) and wR_2 was 0.2201 (all data). The determination of unit cell and intensity data collection was performed using Oxford Diffraction diffractometer at 293(2) K. The crystallographic data (excluding structure factors) for the structures in this manuscript have been deposited with the Cambridge Crystallographic Data Centre as supplementary publication no. CCDC 2070443. Copies of the data can be obtained, without any charge, upon application to CCDC, 12 Union Road, Cambridge CB2 1EZ, UK (fax: + 44 1223 336033 or email: deposit@ccdc.cam.ac.uk).

Refinement model description

Details:

1. Fixed Uiso

At 1.2 times of:

All C(H) groups, All C(H,H) groups, All N(H) groups

2.a Ternary CH refined with riding coordinates:

C007(H007), C009(H009), C00I(H00I)

2.b Secondary CH_2 refined with riding coordinates:

C00J(H00a,H00b), C00O(H00d,H00f), C00P(H00g,H00j)

2.c Aromatic/amide H refined with riding coordinates:

N004(H004), C00C(H00C), C00E(H00E), C00H(H00H), C00K(H00K), C00L(H00L), C00M(H00M), C00Q(H00Q), C00R(H00R), C00S(H00S), C00T(H00T), C00U(H00U), C00V(H00V), C00W(H00W)

Table S1. Crystallographic parameters of the spirooxindole **23f**.

Identification code	**spirooxindole 16**
CCDC No.	198676
Empirical formula	$C_{27}H_{23}BrN_2O_2$
Formula weight	486.0943
Temperature/K	*293(2)*
Crystal system	*trigonal*
Space group	*R-3*
a/Å	*22.6902(11)*
b/Å	*22.6902(11)*
c/Å	*25.8577(11)*
α/°	*90*
β/°	*90*

γ/°	*120*
Volume/Å^3	*11529.1(12)*
Z	*107*
ρ_{calc}g/cm^3	*1.419*
μ/mm^{-1}	*1.637*
F(000)	*5040.0*
Crystal size/mm^3	*0.5 × 0.5 × 0.5*
Radiation	*MoKα (λ = 0.71073)*
2Θ range for data collection/°	*7.814 to 49.99*
Index ranges	*-26 ≤ h ≤ 26, -26 ≤ k ≤ 26, -30 ≤ l ≤ 30*
Reflections collected	*46600*
Independent reflections	*4512 [R_{int} = 0.0710, R_{sigma} = 0.0424]*
Data/restraints/parameters	*4512/0/334*
Goodness-of-fit on F^2	*1.390*
Final R indexes [I>=2σ (I)]	*R_1 = 0.0927, wR_2 = 0.2075*
Final R indexes [all data]	*R_1 = 0.1210, wR_2 = 0.2201*
Largest diff. peak/hole / e Å^{-3}	*0.47/-0.34*

Table S2 Fractional Atomic Coordinates (×10^4) and Equivalent Isotropic Displacement Parameters (Å^2×10^3) for spirooxindole **23f**. U_{eq} is defined as 1/3 of of the trace of the orthogonalised U_{IJ} tensor.

**Atom**	***x***	***y***	***z***	**U(eq)**
Br01	4990.8(4)	4161.1(4)	6011.4(2)	74.7(4)
O002	4548.2(19)	3272.5(17)	3124.7(12)	46.8(9)
O003	3286.9(19)	3567.0(19)	4530.6(14)	48.5(9)
N004	5175(2)	4132.2(18)	3694.8(15)	32.7(9)
N005	4291.2(19)	2462.1(18)	4074.0(16)	32.9(9)
C006	5199(2)	4220(2)	4241.6(18)	28.7(10)
C007	3202(2)	2252(2)	4361.7(18)	32.5(11)
C008	4679(2)	3642(2)	4470.9(17)	29.3(10)
C009	3524(2)	2865(2)	3995.3(17)	30.4(11)
C00A	4284(2)	3110(2)	4052.6(16)	27.6(10)
C00B	4679(2)	3501(2)	3559.8(18)	31.6(11)
C00C	4603(3)	3621(3)	5000.2(19)	36.3(12)
C00D	3309(2)	3388(2)	4091(2)	33.3(11)
C00E	5584(3)	4753(3)	5048(2)	45.2(14)
C00F	5065(3)	4178(3)	5282(2)	40.9(13)
C00G	2437(2)	1829(2)	4339.7(19)	35.9(12)
C00H	5661(3)	4773(3)	4520(2)	39.9(12)

C00I	3604(2)	1899(2)	4225(2)	39.5(12)
C00J	4799(3)	2417(3)	4405(2)	43.6(13)
C00K	2046(3)	1962(3)	4680(2)	44.4(13)
C00L	2099(3)	1302(3)	3990(2)	55.2(16)
C00M	1351(3)	1599(3)	4669(3)	60.4(18)
C00N	3103(3)	3660(3)	3651(2)	51.7(15)
C00O	4424(3)	2000(3)	4868(2)	53.5(15)
C00P	3718(3)	1505(3)	4644(3)	64.9(18)
C00Q	1015(3)	1083(4)	4322(3)	66.3(19)
C00R	1395(4)	936(4)	3981(3)	74(2)
C00S	3234(4)	3579(4)	3136(3)	73(2)
C00T	2768(6)	4012(5)	3751(4)	109(3)
C00U	3015(6)	3833(5)	2735(3)	106(3)
C00V	2696(7)	4182(6)	2852(5)	135(4)
C00W	2541(9)	4250(7)	3342(6)	164(6)
C5	5986(19)	3220(20)	5720(20)	303(17)
C6	6086(16)	2608(12)	5705(16)	283(14)
C2	6401(8)	3479(10)	3800(7)	177(6)
C4	6041(10)	2690(13)	3284(11)	226(8)
C7	6219(15)	3100(20)	6132(13)	307(11)

Table S3 Anisotropic Displacement Parameters (Å^2×10^3) for spirooxindole **23f**. The Anisotropic displacement factor exponent takes the form: $-2\pi^2[h^2a^{*2}U_{11}+2hka^*b^*U_{12}+\ldots]$.

Atom	U_{11}	U_{22}	U_{33}	U_{23}	U_{13}	U_{12}
Br01	83.9(6)	98.6(6)	32.6(4)	-11.9(3)	-2.3(3)	38.7(4)
O002	56(2)	40(2)	26.2(19)	-2.5(16)	10.4(16)	10.7(18)
O003	57(2)	54(2)	44(2)	-15.9(18)	1.3(18)	34(2)
N004	36(2)	22(2)	30(2)	4.9(16)	10.0(17)	6.9(19)
N005	29(2)	23(2)	44(2)	3.1(17)	4.9(18)	11.0(17)
C006	27(2)	27(2)	32(2)	-3(2)	5(2)	13(2)
C007	33(3)	27(2)	29(2)	-1(2)	0(2)	9(2)
C008	33(3)	26(2)	29(2)	-2.0(19)	4(2)	15(2)
C009	34(3)	23(2)	27(2)	-0.1(19)	4(2)	9(2)

C00A	34(3)	21(2)	23(2)	-2.7(18)	4.3(19)	10(2)
C00B	34(3)	35(3)	28(3)	0(2)	5(2)	19(2)
C00C	35(3)	35(3)	34(3)	0(2)	1(2)	14(2)
C00D	32(3)	23(2)	42(3)	-1(2)	4(2)	11(2)
C00E	36(3)	47(3)	47(3)	-22(3)	-7(2)	16(3)
C00F	44(3)	52(3)	30(3)	-8(2)	1(2)	27(3)
C00G	35(3)	34(3)	33(3)	15(2)	5(2)	13(2)
C00H	34(3)	31(3)	46(3)	-5(2)	7(2)	10(2)
C00I	32(3)	28(3)	51(3)	4(2)	1(2)	10(2)
C00J	43(3)	36(3)	59(3)	-2(3)	-1(3)	25(3)
C00K	42(3)	42(3)	50(3)	5(3)	2(3)	22(3)
C00L	44(3)	57(4)	42(3)	-5(3)	1(3)	8(3)
C00M	41(4)	62(4)	86(5)	25(4)	18(3)	32(3)
C00N	69(4)	44(3)	52(4)	5(3)	-2(3)	36(3)
C00O	64(4)	60(4)	49(3)	19(3)	5(3)	40(3)
C00P	55(4)	49(4)	93(5)	38(3)	24(3)	27(3)
C00Q	34(3)	70(5)	89(5)	25(4)	-5(4)	21(3)
C00R	56(4)	64(4)	67(4)	-1(3)	-20(4)	3(4)
C00S	101(6)	80(5)	55(4)	17(4)	3(4)	58(5)
C00T	166(9)	138(8)	88(6)	-5(6)	-7(6)	125(8)
C00U	159(9)	117(7)	72(5)	26(5)	-8(5)	91(7)
C00V	201(12)	155(10)	116(9)	19(8)	-32(9)	138(10)
C00W	277(17)	224(14)	126(10)	-8(9)	-43(10)	227(14)
C5	230(30)	190(30)	500(50)	0(30)	-120(30)	110(30)
C6	200(30)	180(20)	480(40)	-90(30)	-30(20)	98(19)
C2	124(14)	148(15)	212(13)	6(13)	57(11)	33(8)
C4	127(15)	240(20)	295(19)	24(17)	-11(14)	82(10)
C7	280(20)	300(40)	360(30)	100(30)	105(19)	160(30)

Table S4 Bond Lengths for spirooxindole **23f.**

Atom	Atom	Length/Å	Atom	Atom	Length/Å
Br01	C00F	1.893(5)	C00L	C00R	1.384(9)
O002	C00B	1.212(5)	C00M	C00Q	1.366(10)

O003	C00D	1.216(6)	C00N	C00S	1.398(9)
N004	C006	1.425(6)	C00N	C00T	1.375(10)
N004	C00B	1.351(6)	C00O	C00P	1.537(10)
N005	C00A	1.480(6)	C00Q	C00R	1.384(10)
N005	C00I	1.492(6)	C00S	C00U	1.393(10)
N005	C00J	1.480(6)	C00T	C00W	1.397(15)
C006	C008	1.385(6)	C00U	C00V	1.347(14)
C006	C00H	1.369(7)	C00V	C00W	1.345(17)
C007	C009	1.532(6)	C5	C6[1]	1.42(3)
C007	C00G	1.506(7)	C5	C6	1.53(3)
C007	C00I	1.528(7)	C5	C7	1.29(3)
C008	C00A	1.531(6)	C6	C5[2]	1.42(3)
C008	C00C	1.377(6)	C6	C7	1.48(4)
C009	C00A	1.532(7)	C6	C7[2]	1.96(5)
C009	C00D	1.511(7)	C2	C2[1]	1.42(3)
C00A	C00B	1.555(6)	C2	C2[2]	1.42(3)
C00C	C00F	1.379(7)	C2	C4[3]	1.31(3)
C00D	C00N	1.478(8)	C2	C4[1]	1.63(3)
C00E	C00F	1.385(8)	C4	C2[2]	1.63(3)
C00E	C00H	1.376(7)	C4	C2[4]	1.31(3)
C00G	C00K	1.385(7)	C4	C4[3]	1.462(18)
C00G	C00L	1.387(8)	C4	C4[4]	1.462(18)
C00I	C00P	1.507(8)	C7	C6[1]	1.96(5)
C00J	C00O	1.499(8)	C7	C7[2]	1.52(5)
C00K	C00M	1.367(8)	C7	C7[1]	1.52(5)

[1]1+Y-X,1-X,+Z; [2]1-Y,+X-Y,+Z; [3]1/3+Y,2/3-X+Y,2/3-Z; [4]1/3-Y+X,-1/3+X,2/3-Z

Table S5 Bond Angles for spirooxindole **23f.**

Atom	Atom	Atom	Angle/°	Atom	Atom	Atom	Angle/°
C00B	N004	C006	111.0(4)	C00S	C00N	C00D	123.1(5)
C00A	N005	C00I	109.6(3)	C00T	C00N	C00D	118.8(6)
C00J	N005	C00A	119.1(4)	C00T	C00N	C00S	118.1(6)
C00J	N005	C00I	107.8(4)	C00J	C00O	C00P	102.4(5)
C008	C006	N004	109.7(4)	C00I	C00P	C00O	105.2(4)

C00H	C006	N004	127.4(4)	C00M	C00Q	C00R	118.5(6)
C00H	C006	C008	122.9(4)	C00L	C00R	C00Q	121.1(6)
C00G	C007	C009	115.5(4)	C00U	C00S	C00N	120.7(7)
C00I	C007	C009	101.2(4)	C00N	C00T	C00W	120.0(9)
C00I	C007	C00G	117.2(4)	C00V	C00U	C00S	119.1(9)
C006	C008	C00A	109.2(4)	C00W	C00V	C00U	121.7(9)
C00C	C008	C006	119.4(4)	C00V	C00W	C00T	120.1(10)
C00C	C008	C00A	131.4(4)	C6	C5	$C6^{1}$	125(3)
C007	C009	C00A	102.2(4)	C7	C5	C6	63(2)
C00D	C009	C007	114.5(4)	C7	C5	$C6^{1}$	93(3)
C00D	C009	C00A	116.4(4)	C5	C6	$C5^{2}$	115(3)
N005	C00A	C008	117.6(4)	C5	C6	$C7^{2}$	88(2)
N005	C00A	C009	102.2(3)	$C5^{2}$	C6	$C7^{2}$	40.8(18)
N005	C00A	C00B	107.9(3)	C7	C6	C5	50.5(19)
C008	C00A	C009	117.6(4)	C7	C6	$C5^{2}$	90(3)
C008	C00A	C00B	100.7(3)	C7	C6	$C7^{2}$	50(3)
C009	C00A	C00B	110.6(4)	$C2^{2}$	C2	$C2^{1}$	59.997(6)
O002	C00B	N004	126.1(4)	$C4^{3}$	C2	$C2^{2}$	98(2)
O002	C00B	C00A	124.6(4)	$C4^{1}$	C2	$C2^{1}$	83.9(15)
N004	C00B	C00A	109.2(4)	$C4^{1}$	C2	$C2^{2}$	116.0(10)
C00F	C00C	C008	117.9(5)	$C4^{3}$	C2	$C2^{1}$	123.1(11)
O003	C00D	C009	120.0(4)	$C4^{3}$	C2	$C4^{1}$	58.2(10)
O003	C00D	C00N	120.2(4)	$C2^{4}$	C4	$C2^{2}$	119.5(19)
C00N	C00D	C009	119.7(4)	$C2^{4}$	C4	$C4^{3}$	72.0(19)
C00H	C00E	C00F	119.9(5)	$C2^{4}$	C4	$C4^{4}$	95(2)
C00C	C00F	Br01	119.2(4)	$C4^{4}$	C4	$C2^{2}$	49.8(15)
C00C	C00F	C00E	122.2(5)	$C4^{3}$	C4	$C2^{2}$	83(2)
C00E	C00F	Br01	118.6(4)	$C4^{3}$	C4	$C4^{4}$	117.0(13)
C00K	C00G	C007	119.7(5)	C5	C7	C6	66(2)
C00K	C00G	C00L	117.7(5)	C5	C7	$C6^{1}$	46(2)
C00L	C00G	C007	122.6(5)	C5	C7	$C7^{1}$	94(3)
C006	C00H	C00E	117.7(5)	C5	C7	$C7^{2}$	121(3)
N005	C00I	C007	104.7(4)	C6	C7	$C6^{1}$	98(3)

N005	C00I	C00P	106.3(4)	C6	C7	C7[2]	81(3)
C007	C00I	C00P	118.1(5)	C6	C7	C7[1]	119.4(17)
N005	C00J	C00O	106.8(4)	C7[1]	C7	C6[1]	48(2)
C00M	C00K	C00G	121.8(6)	C7[2]	C7	C6[1]	95.5(19)
C00R	C00L	C00G	120.2(6)	C7[2]	C7	C7[1]	60.002(14)
C00K	C00M	C00Q	120.7(6)				

[1]1+Y-X,1-X,+Z; [2]1-Y,+X-Y,+Z; [3]1/3+Y,2/3-X+Y,2/3-Z; [4]1/3-Y+X,-1/3+X,2/3-Z

Table S6 Hydrogen Atom Coordinates (Å×10^4) and Isotropic Displacement Parameters (Å^2×10^3) for spirooxindole **23f.**

Atom	*x*	*y*	*z*	U(eq)
H004	5441	4439.61	3479.51	39
H007	3326.13	2426.7	4715.25	39
H009	3389.63	2693.76	3641.82	37
H00C	4252.31	3242.08	5162.88	44
H00E	5880.91	5124.64	5248.49	54
H00H	6015.06	5148.8	4356.76	48
H00I	3391.61	1600.36	3926.51	47
H00A	5149.97	2868.04	4511.19	52
H00B	5011.95	2201.89	4218.66	52
H00K	2262.72	2306.6	4923.52	53
H00L	2345.68	1193.89	3759.65	66
H00M	1103.36	1704.44	4900.32	73
H00D	4394.42	2281.45	5137.89	64
H00F	4640.68	1758.31	5006.45	64
H00G	3709.47	1104.91	4501.96	78
H00J	3370.7	1362.26	4908.96	78
H00Q	542.12	836.11	4313.71	80
H00R	1173.36	584.69	3743.43	89
H00S	3470.94	3353.01	3059.3	87
H00T	2693.07	4092.89	4090.74	131
H00U	3087.54	3762.82	2392.49	128
H00V	2580.17	4381.84	2586.07	162
H00W	2281.61	4454.38	3408.95	196

^{1}H NMR and ^{13}C NMR Spectral Data of 23a-f, 24a-f and 25a-g.

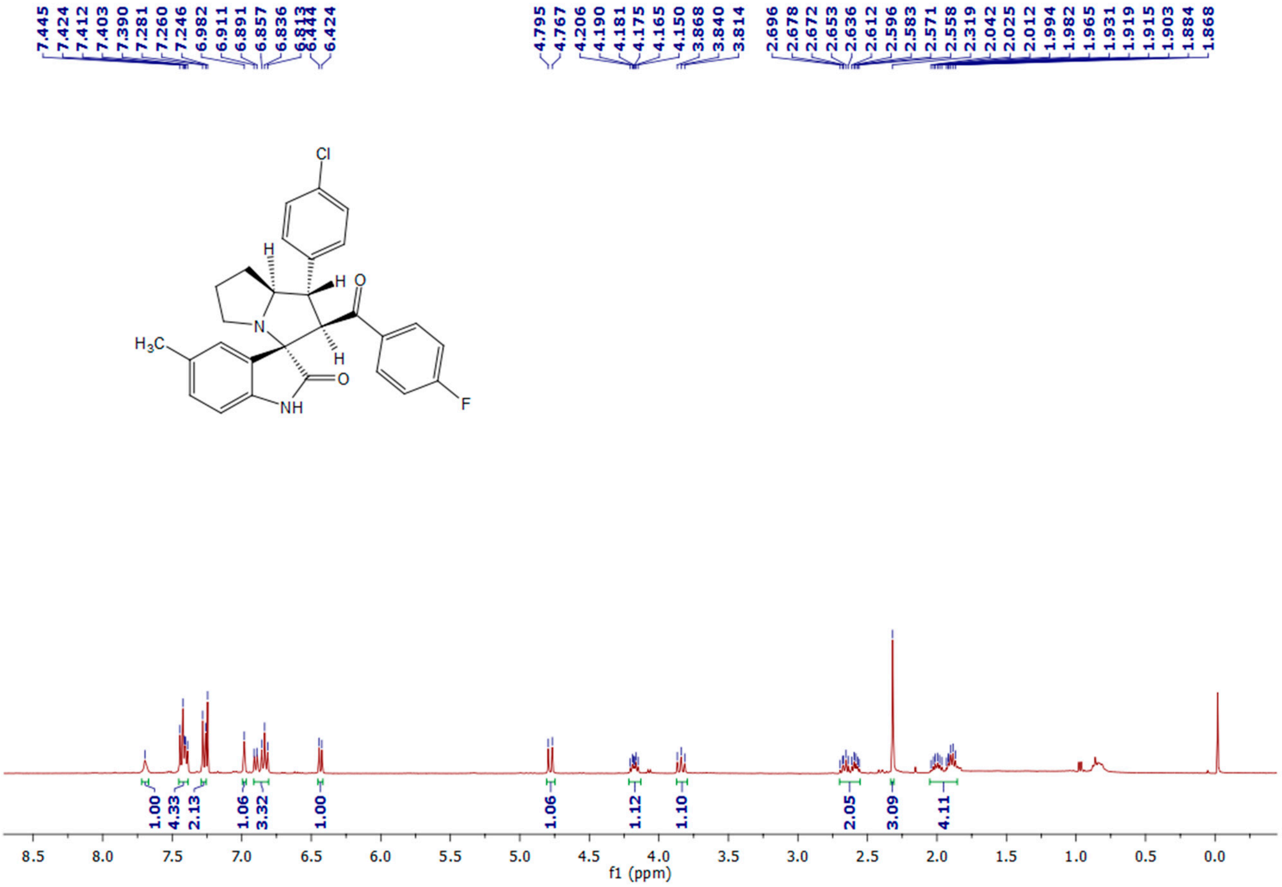

Figure S1: ^{1}H-NMR spectra of compound **23a**.

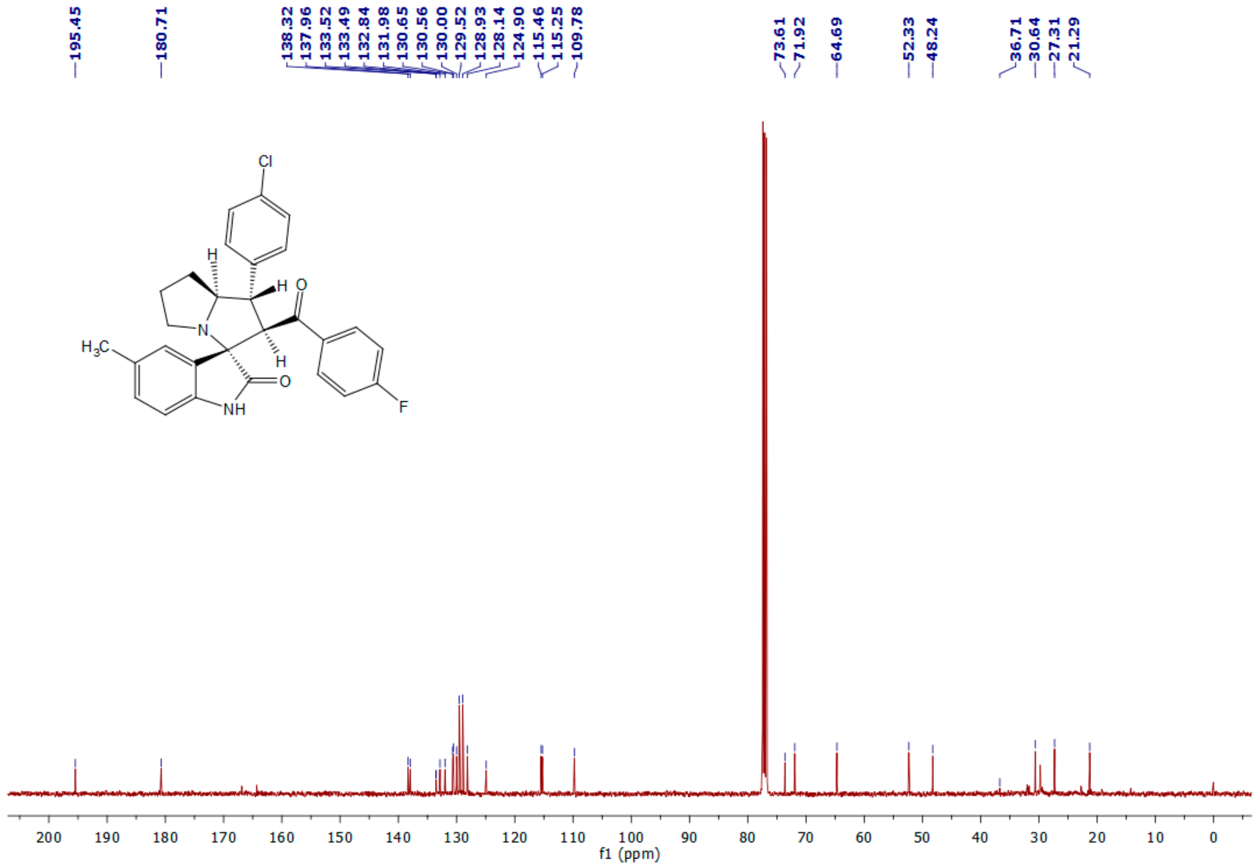

Figure S2: ^{13}C-NMR spectra of compound **23a**.

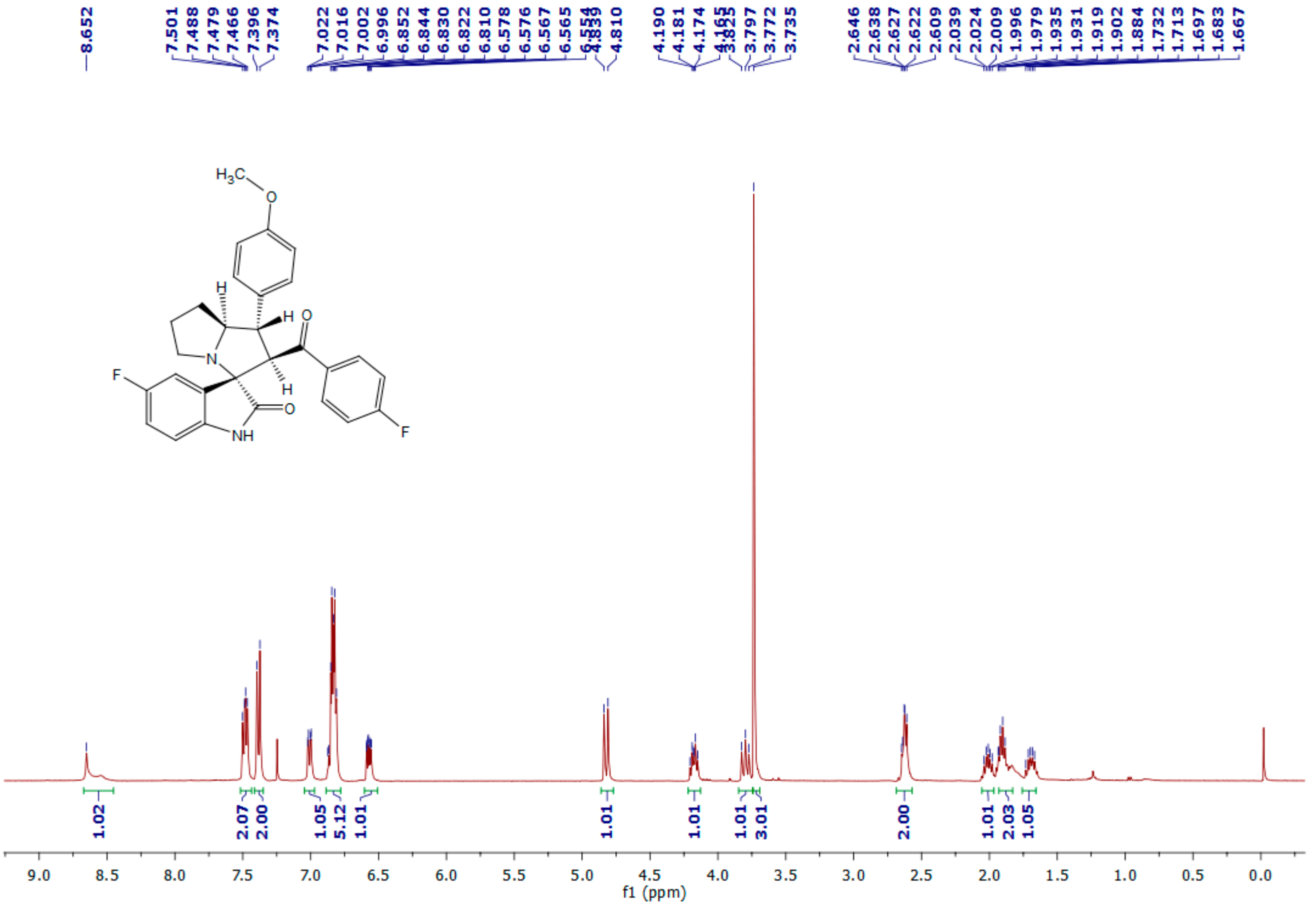

Figure S3: ^{1}H-NMR spectra of compound **23b**.

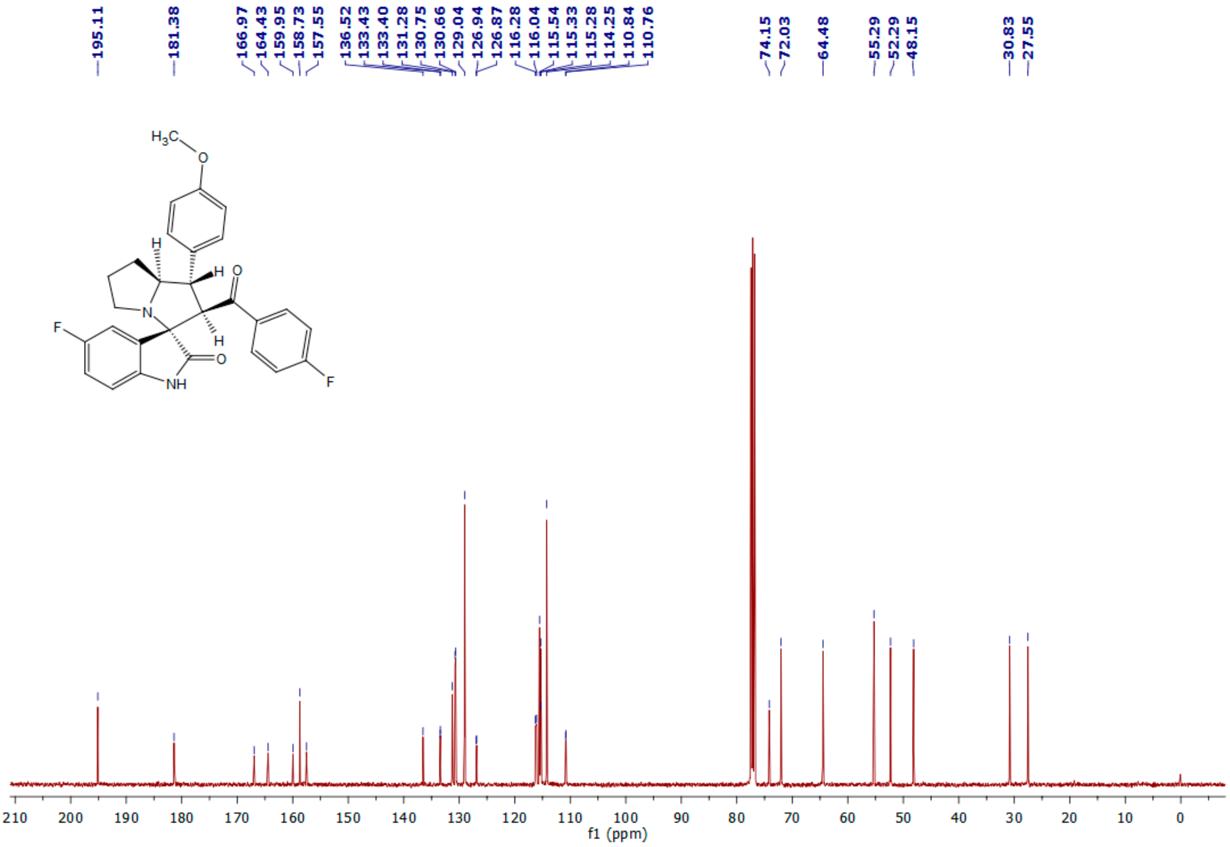

Figure S4: ^{13}C-NMR spectra of compound **23b**.

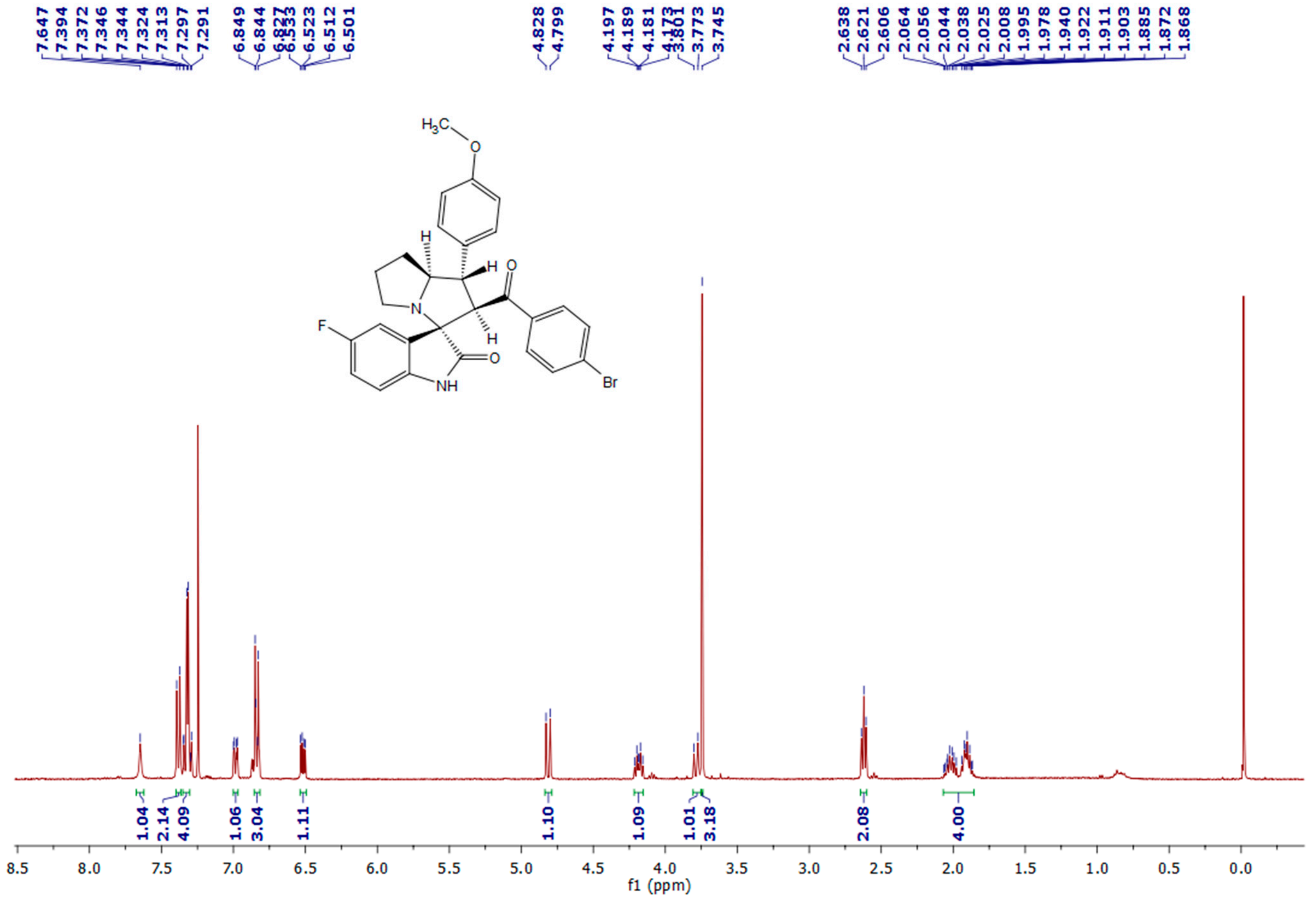

Figure S5: ^{1}H-NMR spectra of compound **23c**.

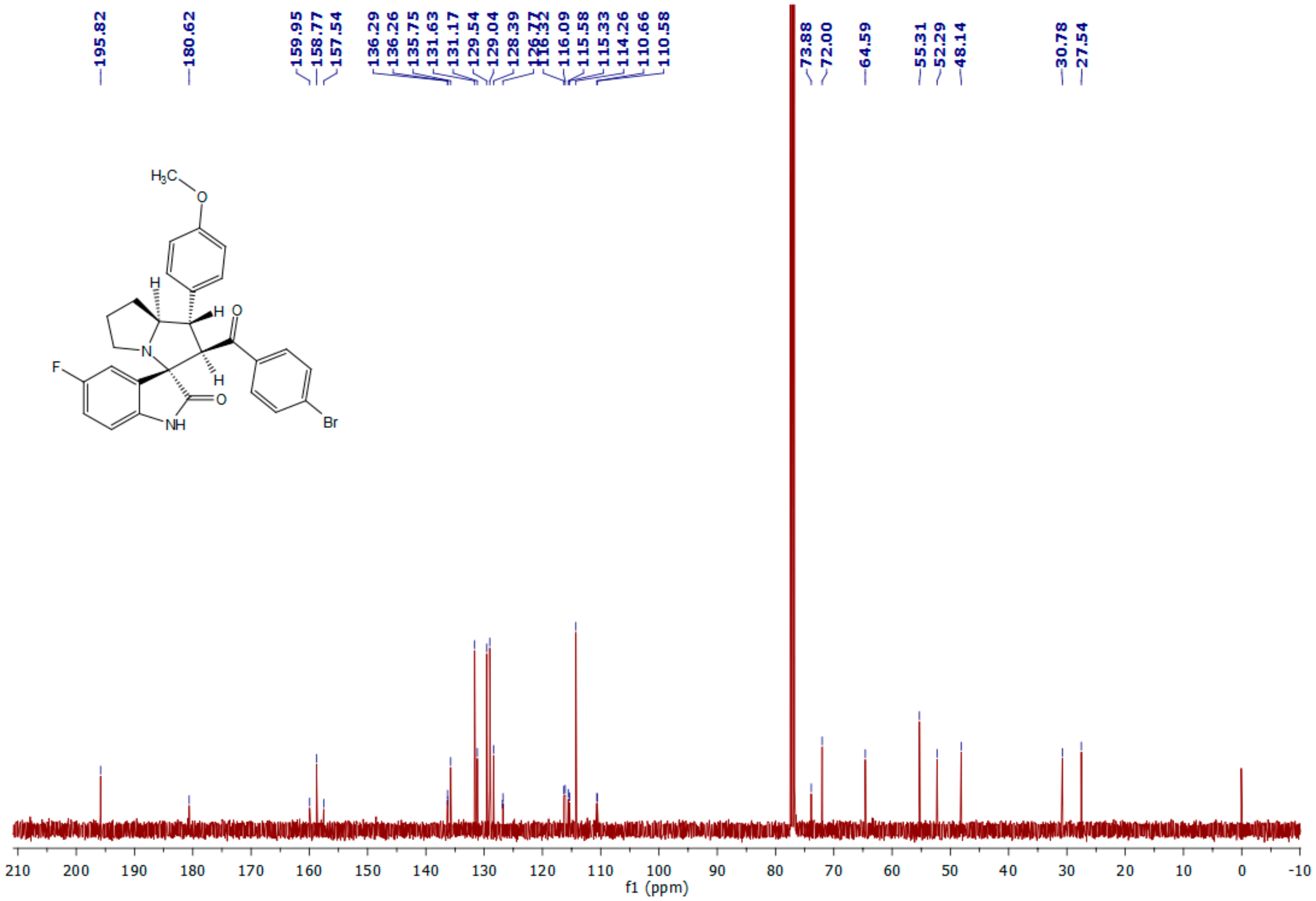

Figure S6: ^{13}C-NMR spectra of compound **23c**.

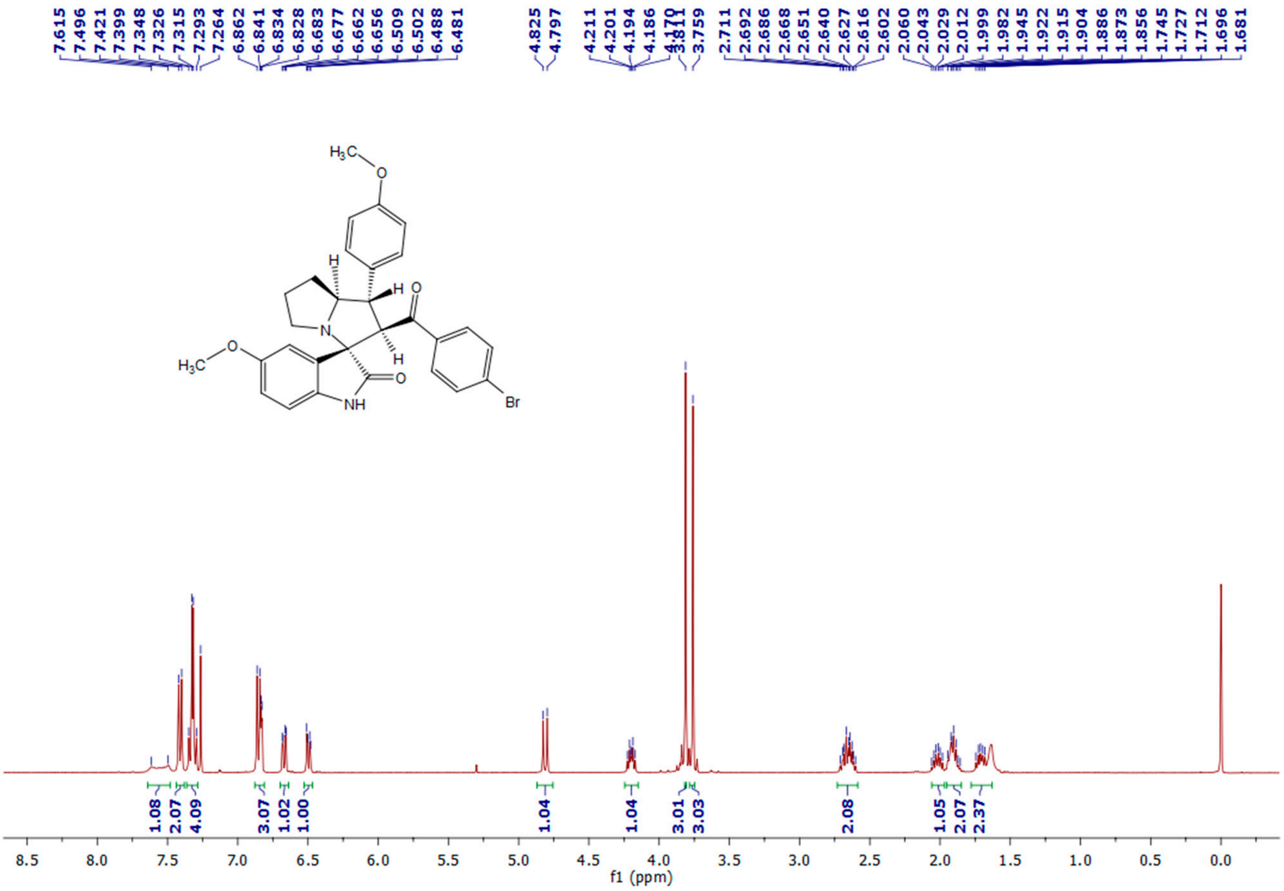

Figure S7: ^{1}H-NMR spectra of compound **23d**.

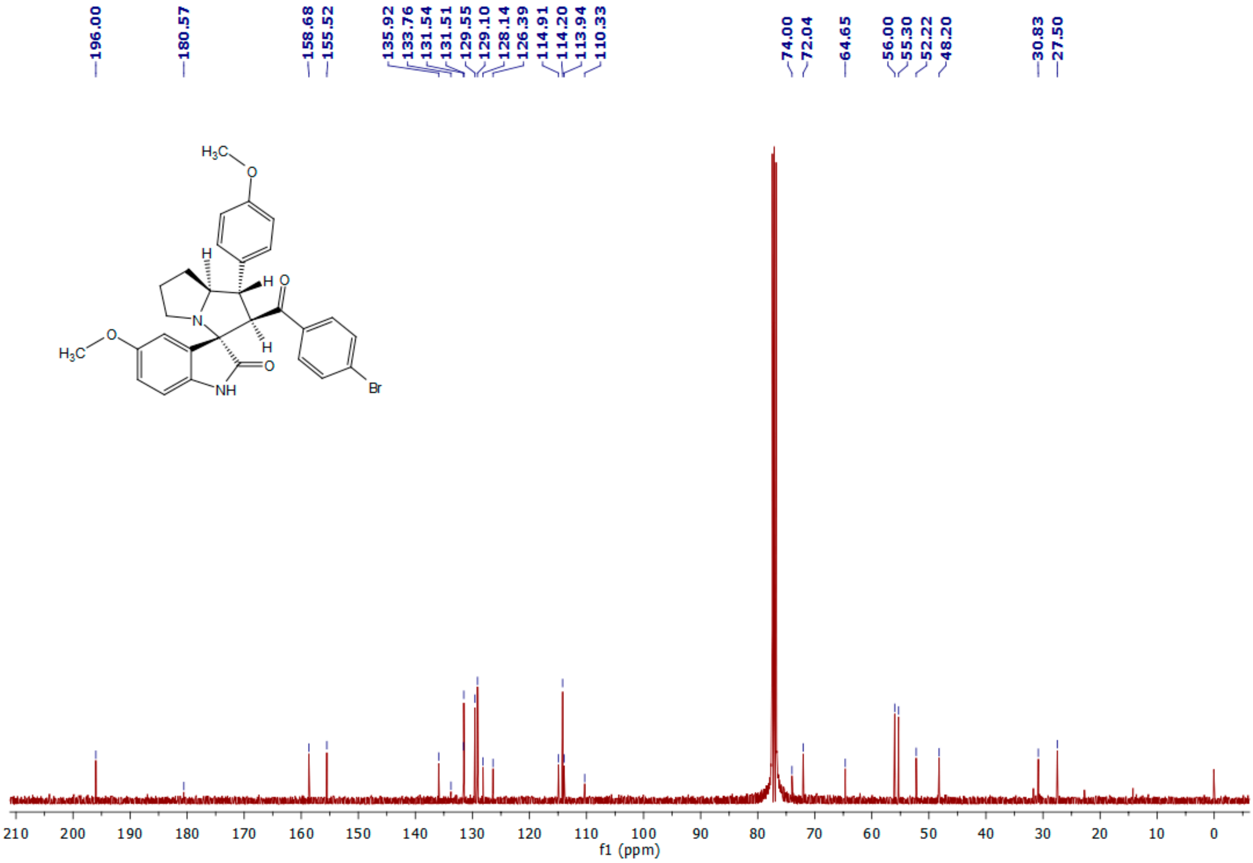

Figure S8: ^{13}C-NMR spectra of compound **23d**.

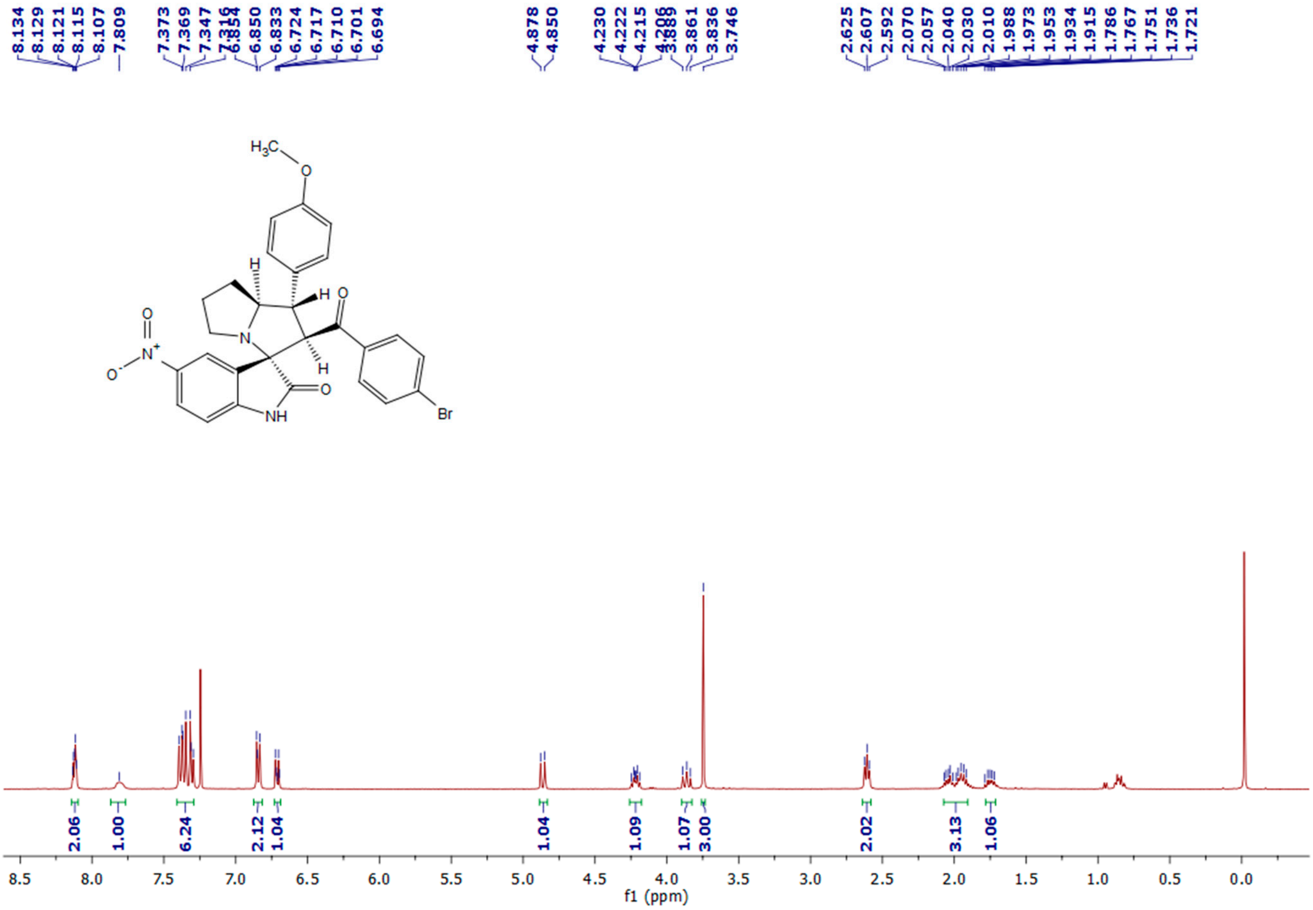

Figure S9: ^{1}H-NMR spectra of compound **23e**.

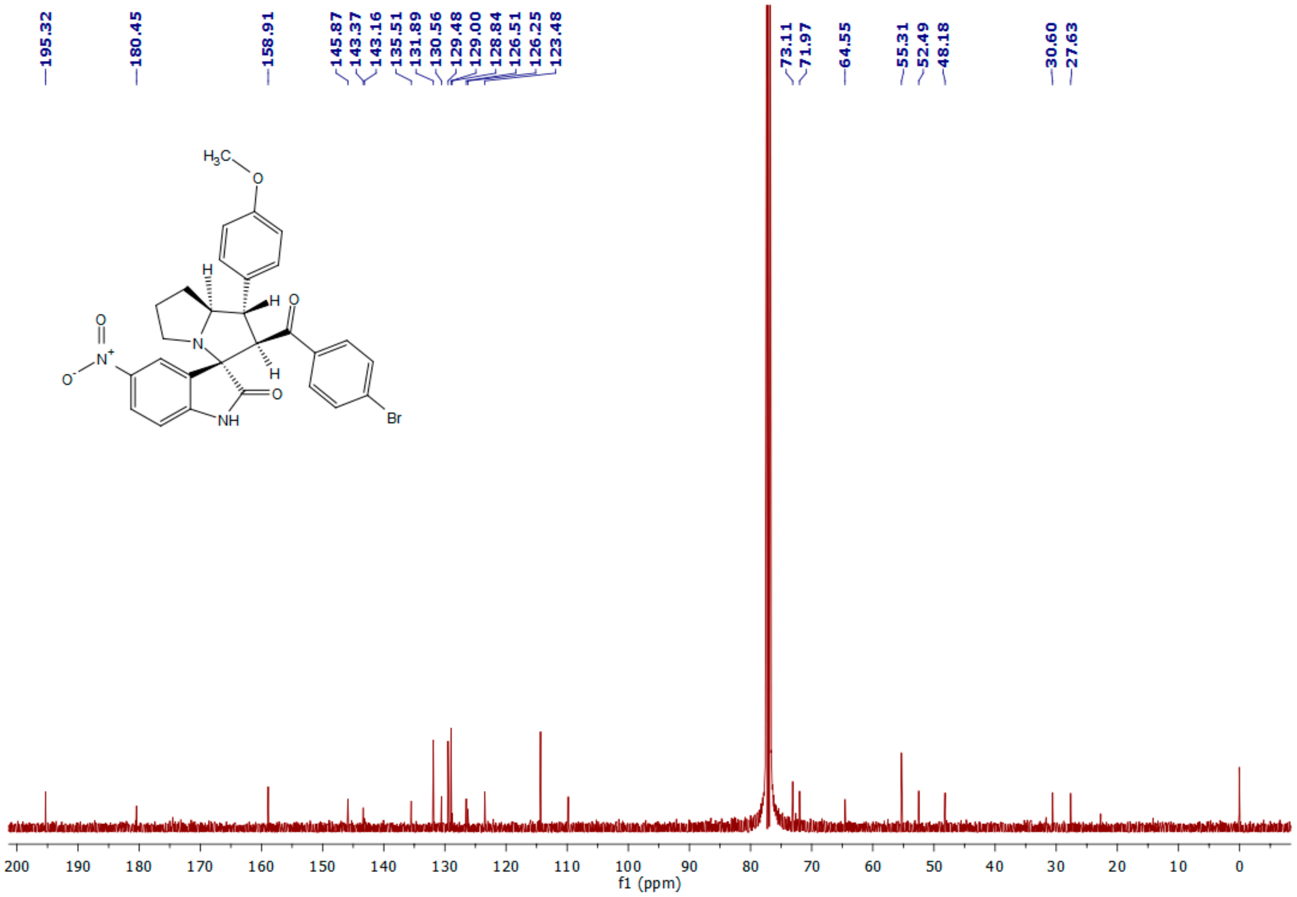

Figure S10: ^{13}C-NMR spectra of compound **23e**.

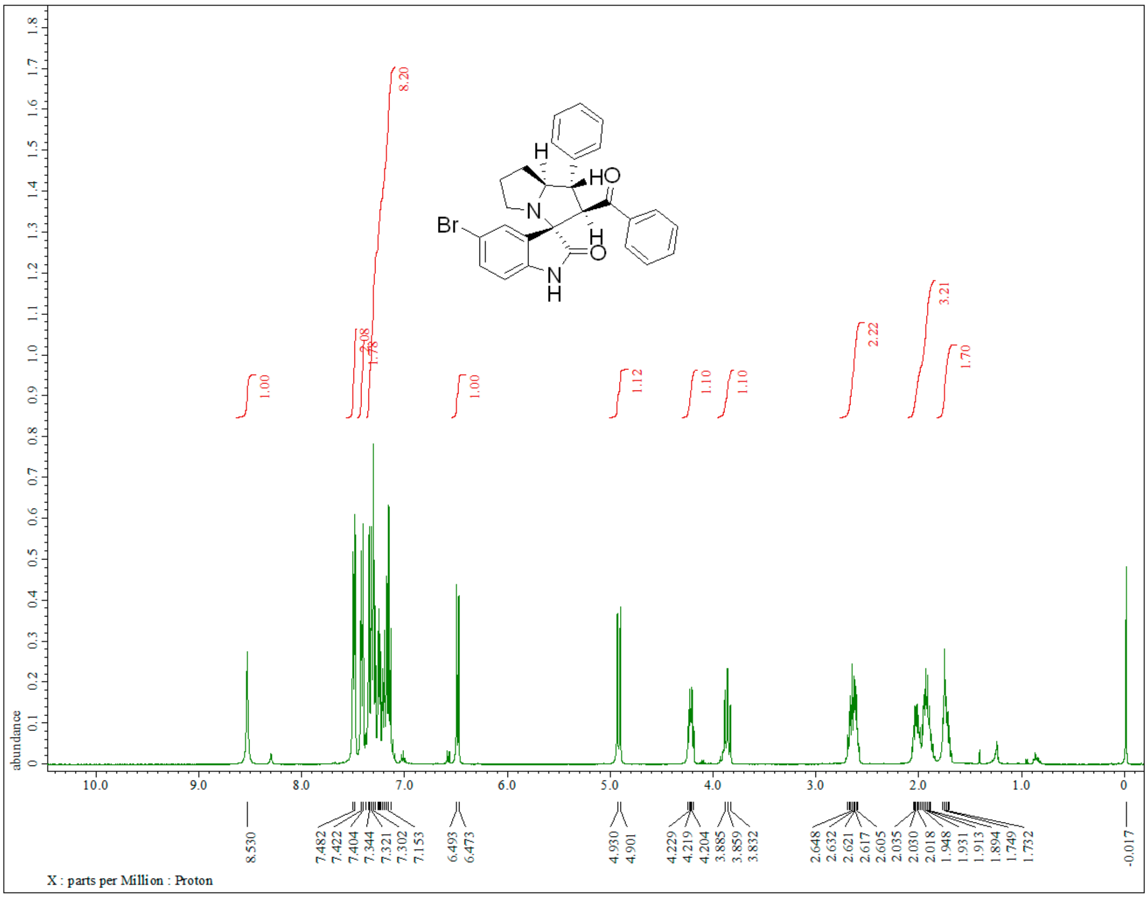

Figure S11.^{1}H-NMR spectrum of the **23f**.

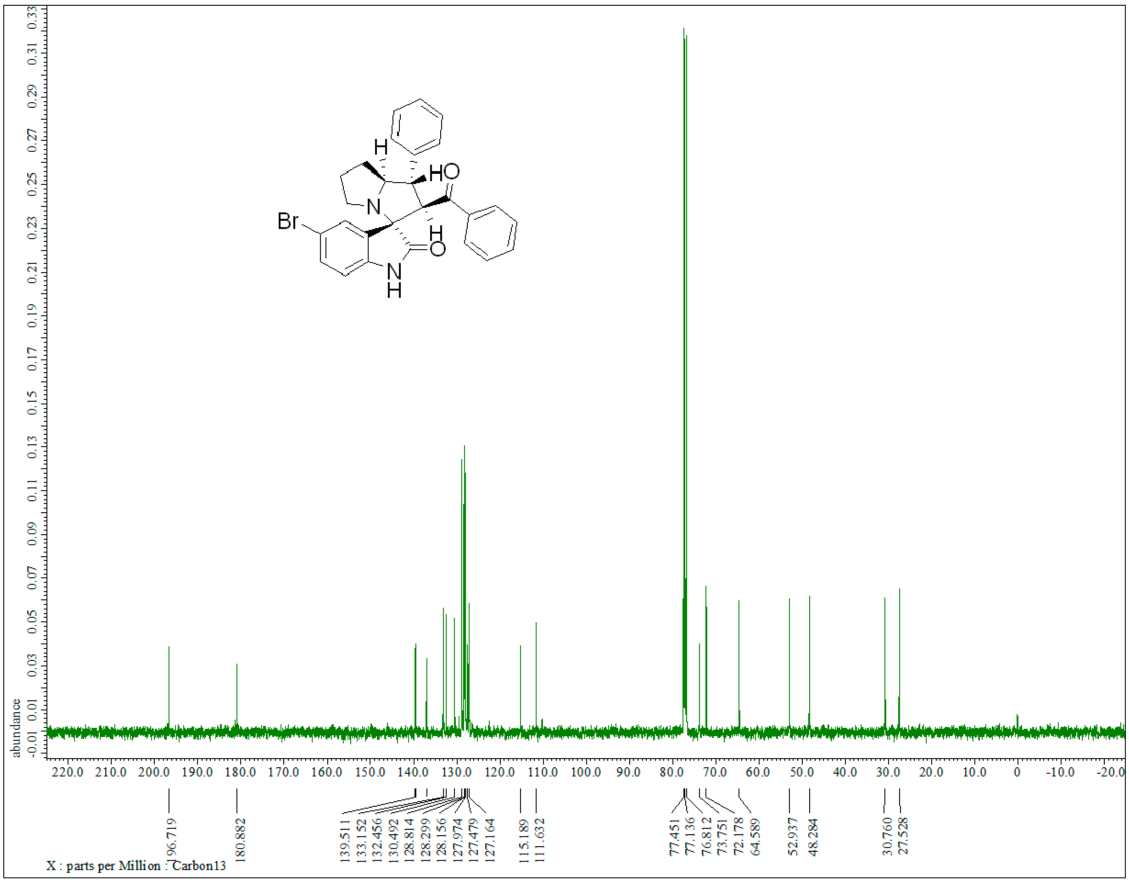

Figure S12.^{13}C-NMR spectrum of the **23f**.

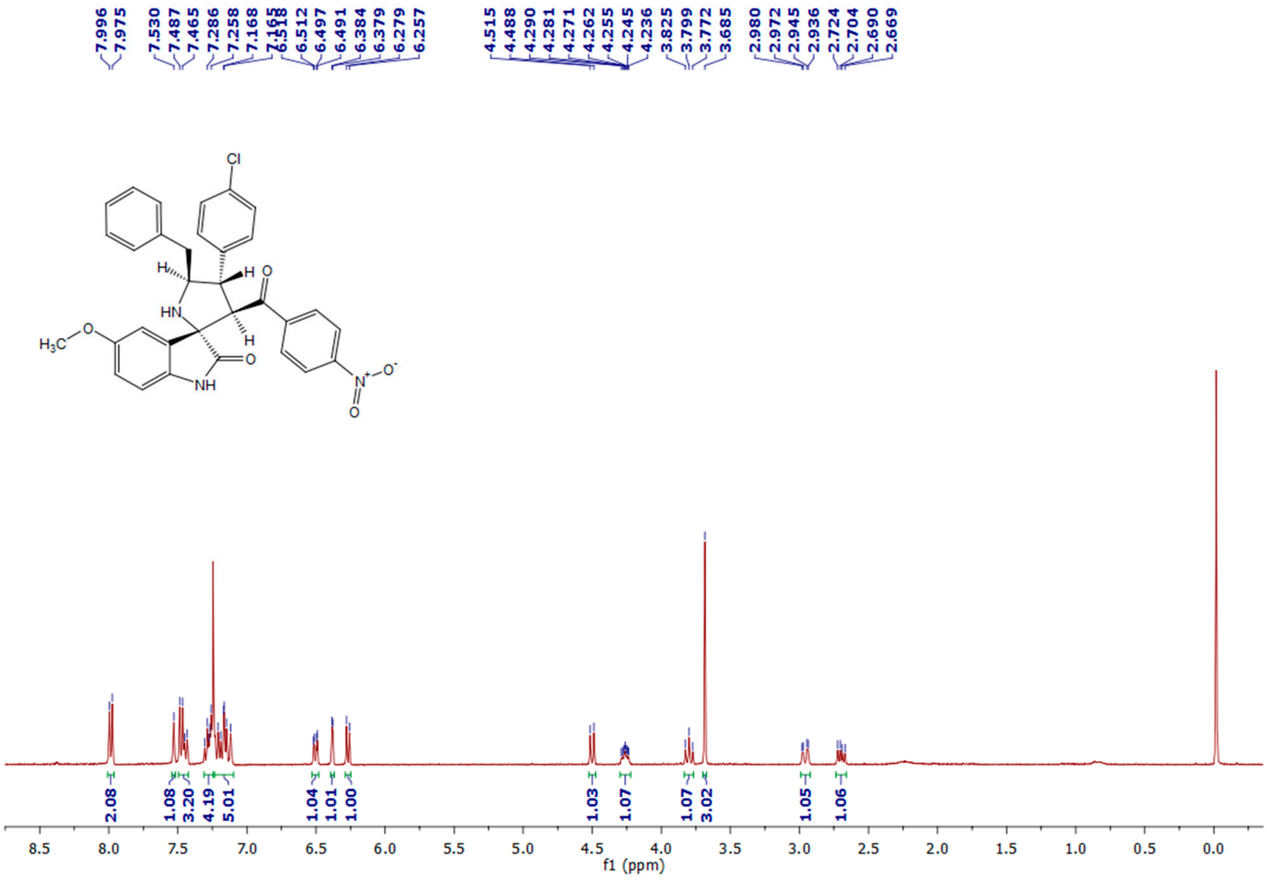

Figure S13: ^{1}H-NMR spectra of compound **24a**.

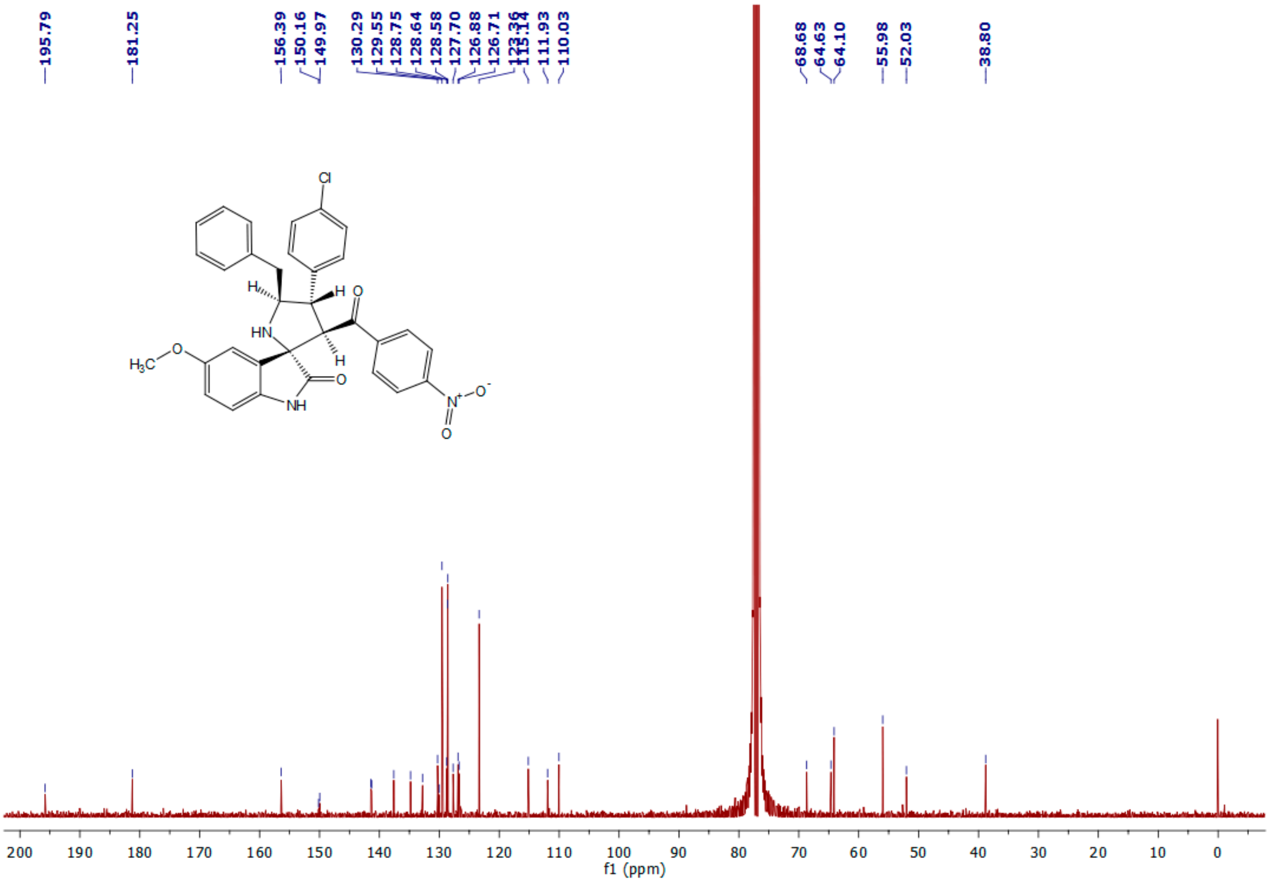

Figure S14: ^{13}C-NMR spectra of compound **24a**.

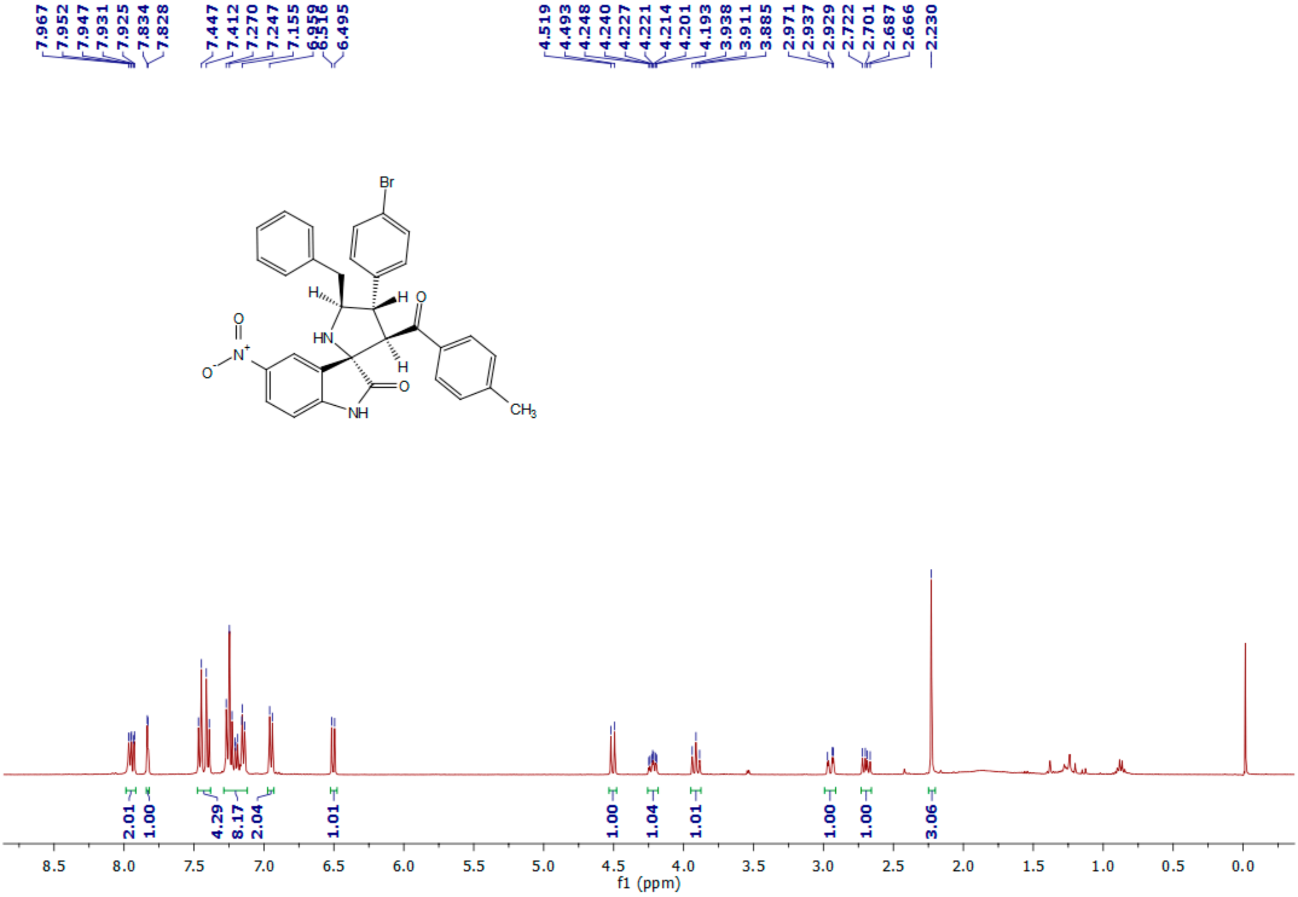

Figure S15: ^{1}H-NMR spectra of compound **24b**.

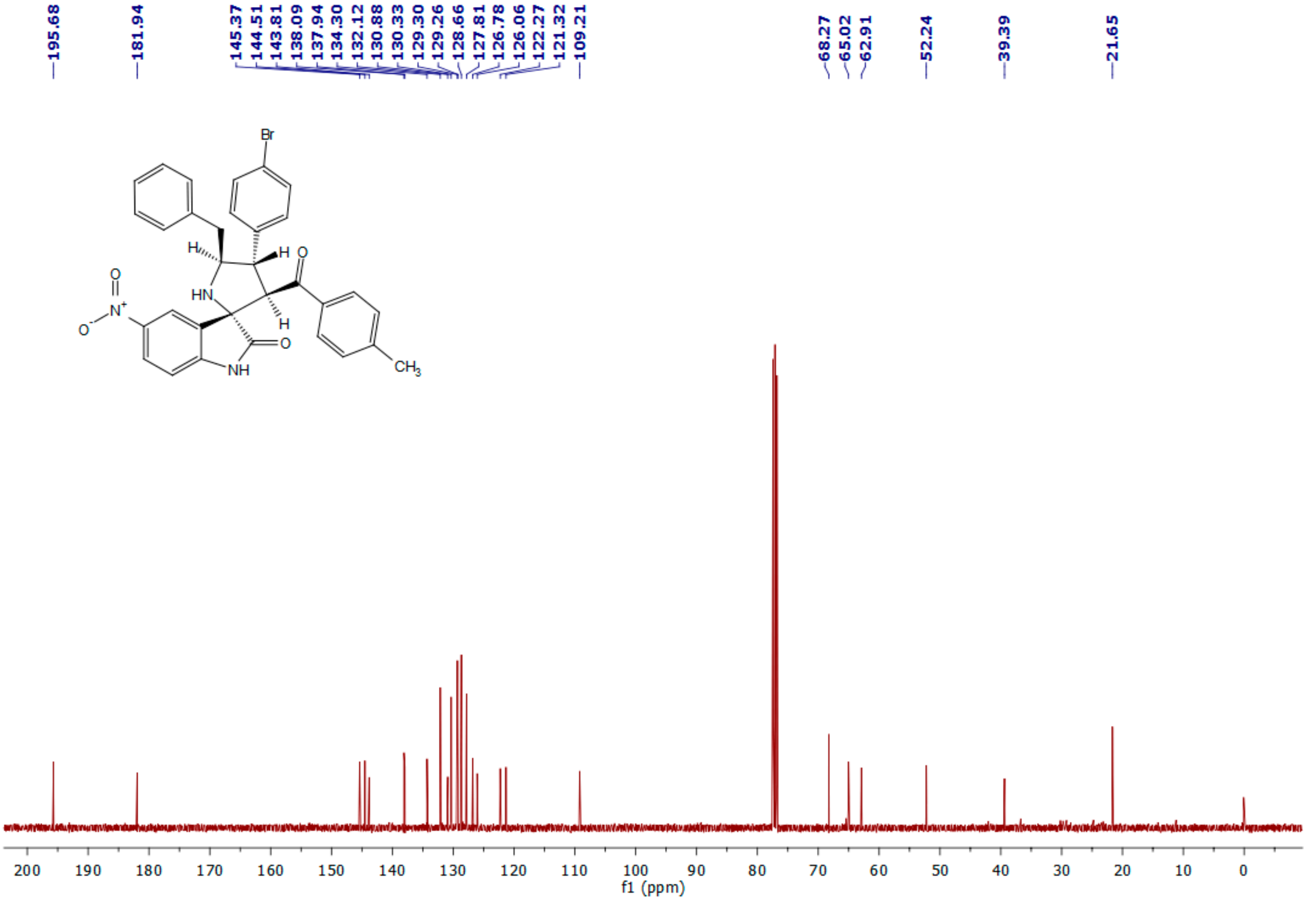

Figure S16: ^{13}C-NMR spectra of compound **24b**.

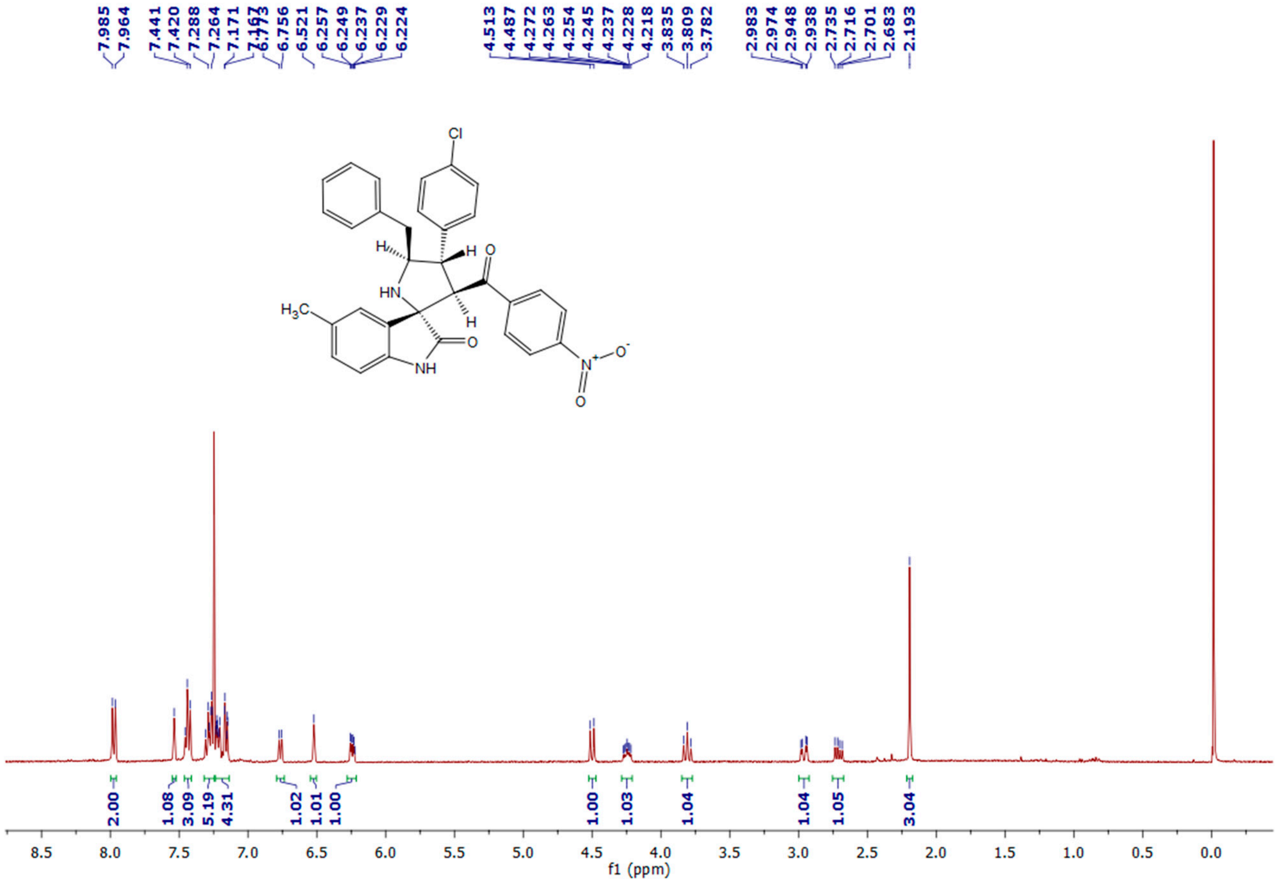

Figure S17: ^{1}H-NMR spectra of compound **24c**.

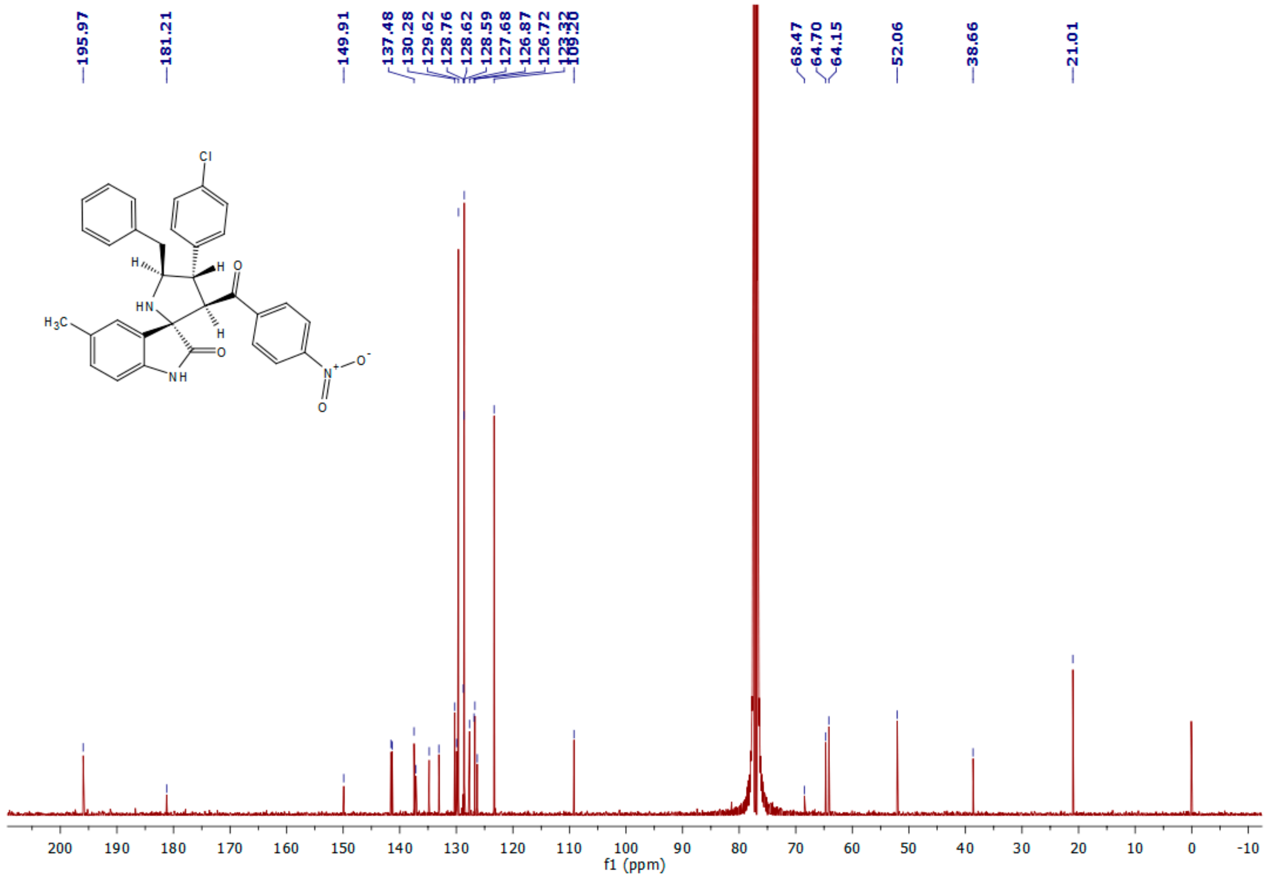

Figure S18: ^{13}C-NMR spectra of compound **24c**.

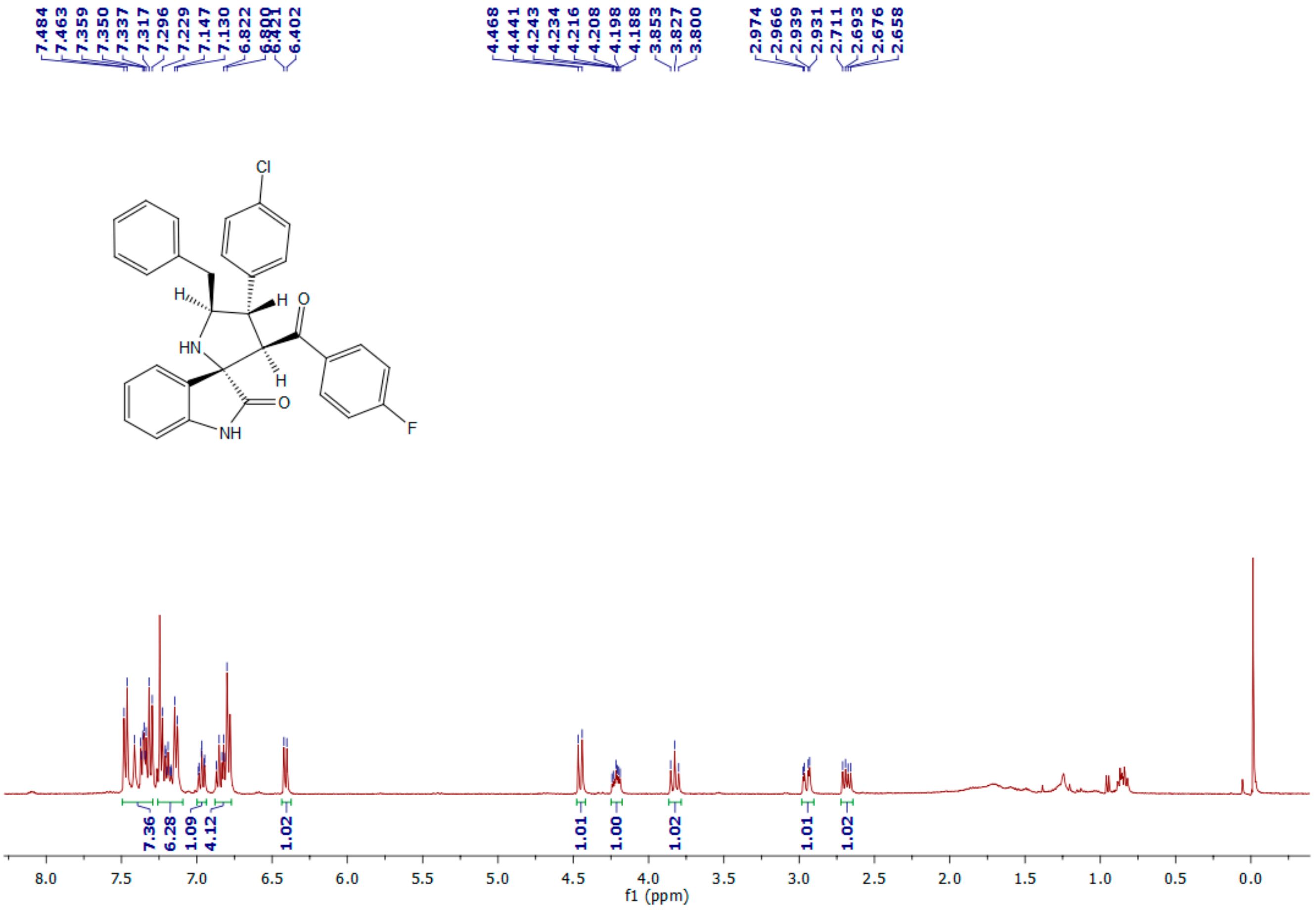

Figure S19: ^{1}H-NMR spectra of compound **24d**.

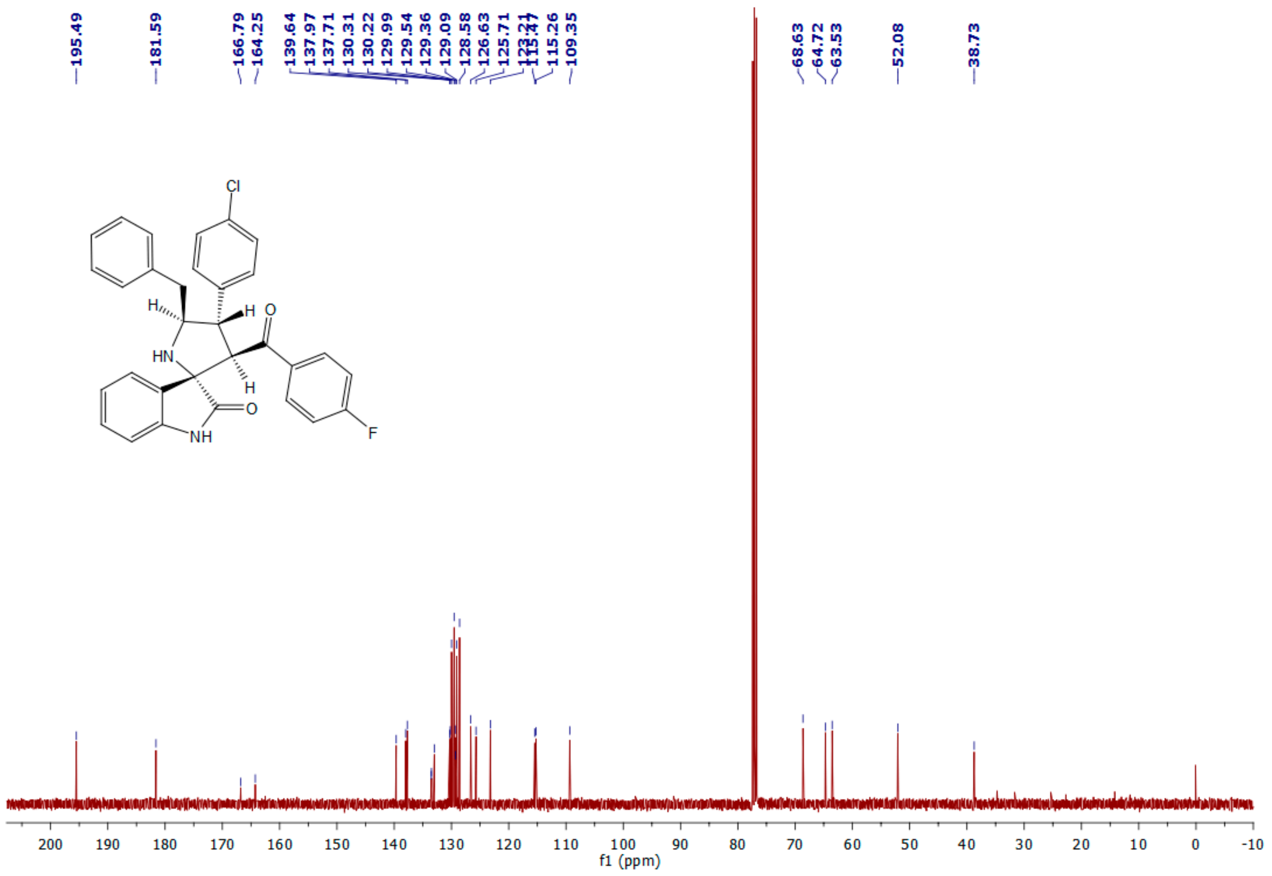

Figure S20: ^{13}C-NMR spectra of compound **24d**.

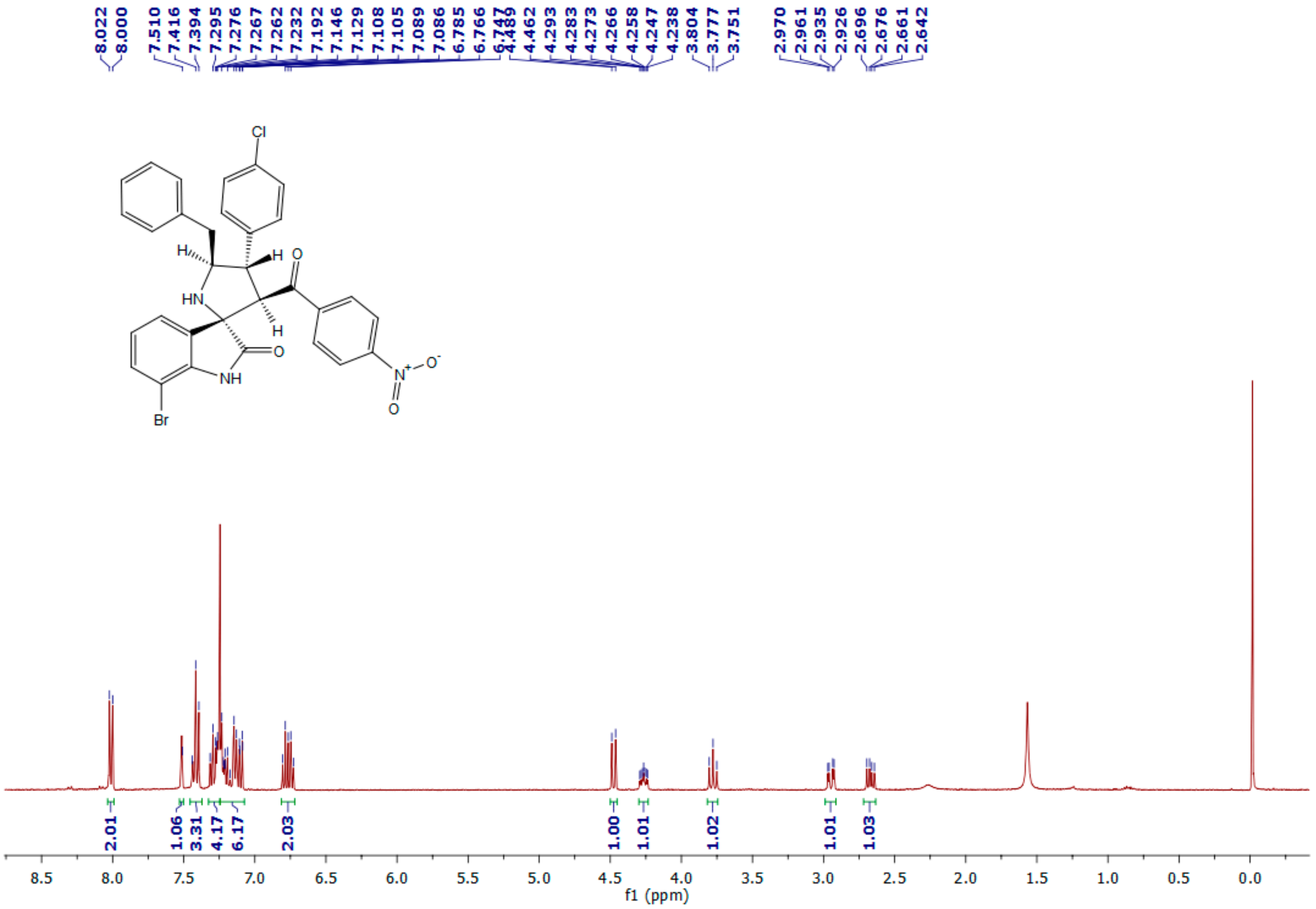

Figure S21: ^{1}H-NMR spectra of compound **24e**.

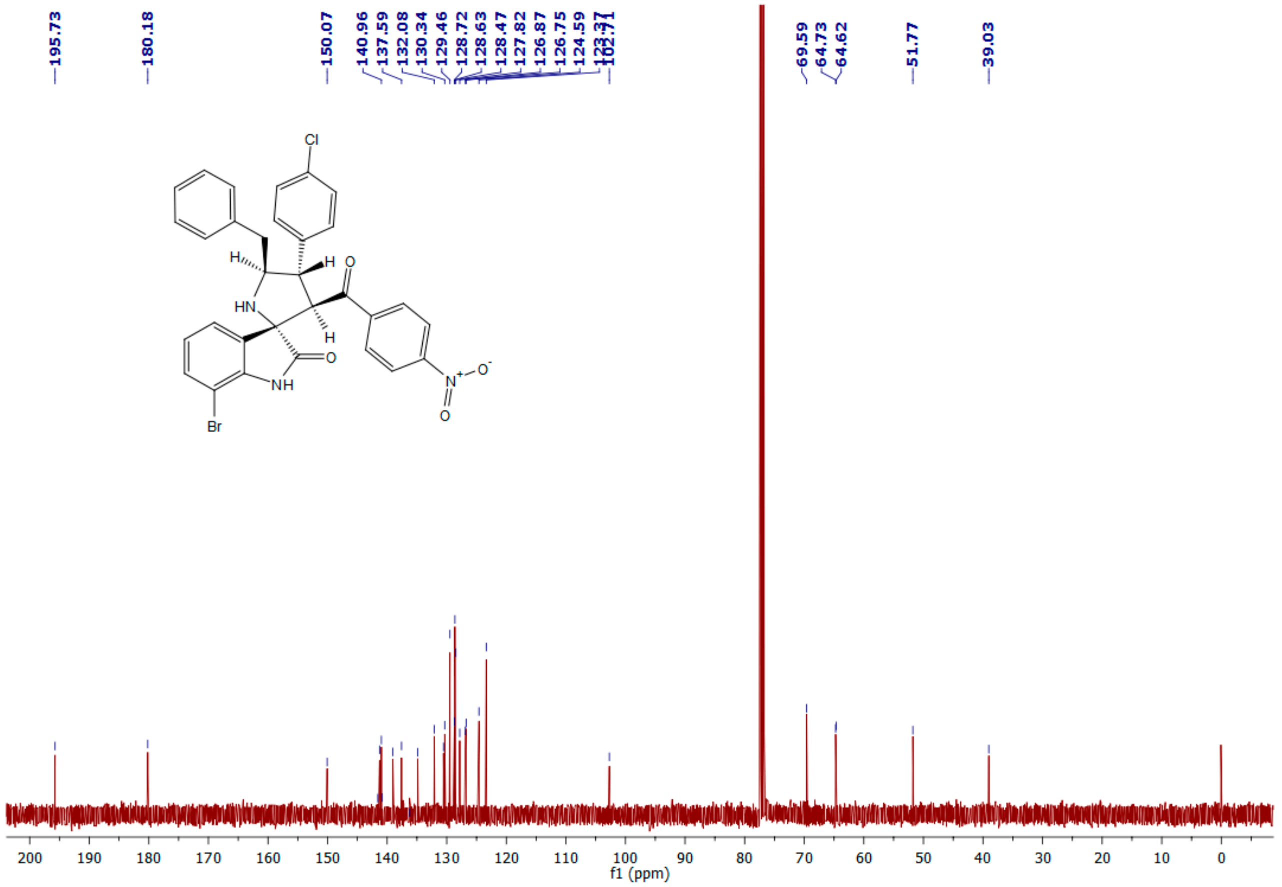

Figure S22: ^{13}C-NMR spectra of compound **24e**.

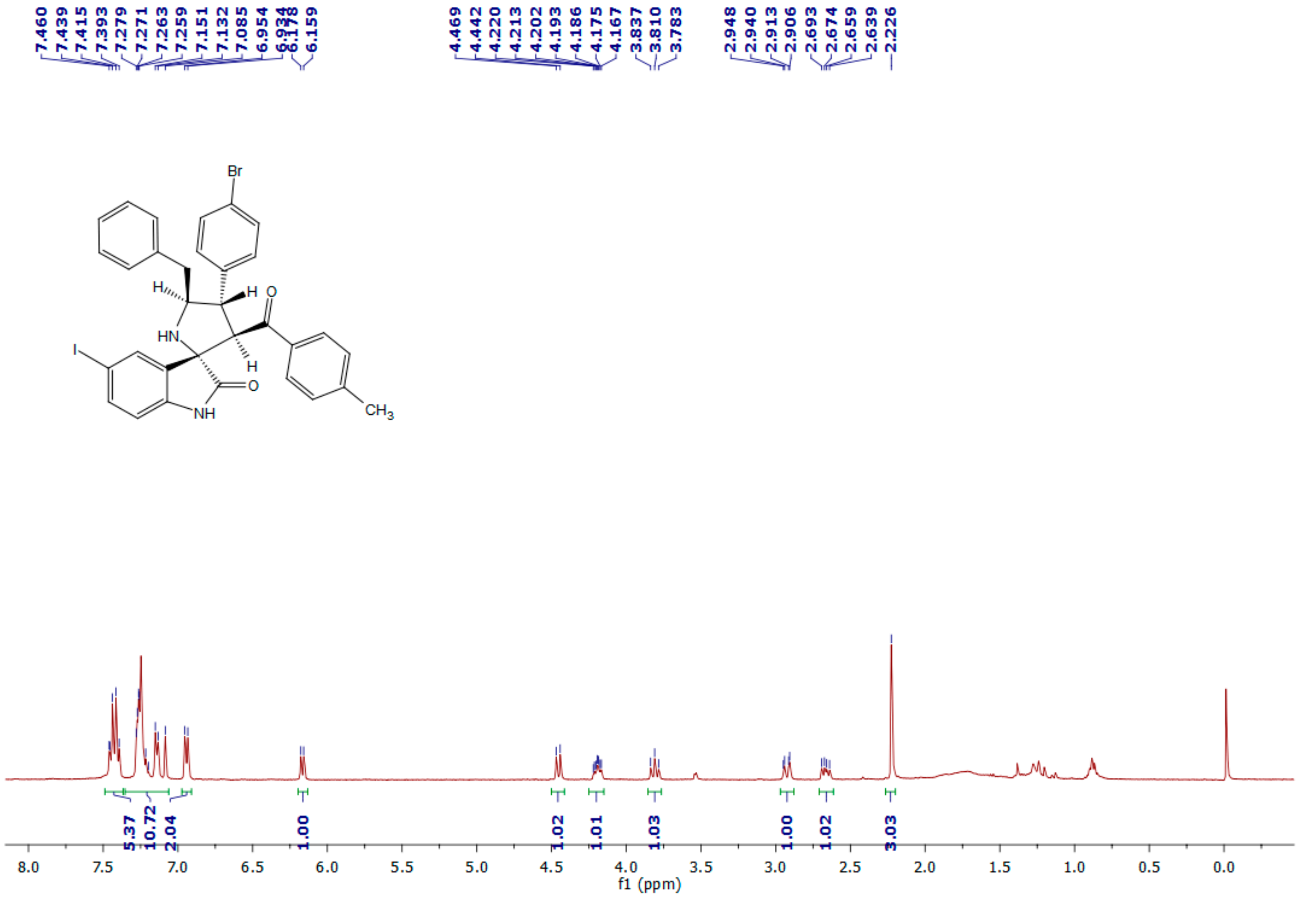

Figure S23: ^{1}H-NMR spectra of compound **24f**.

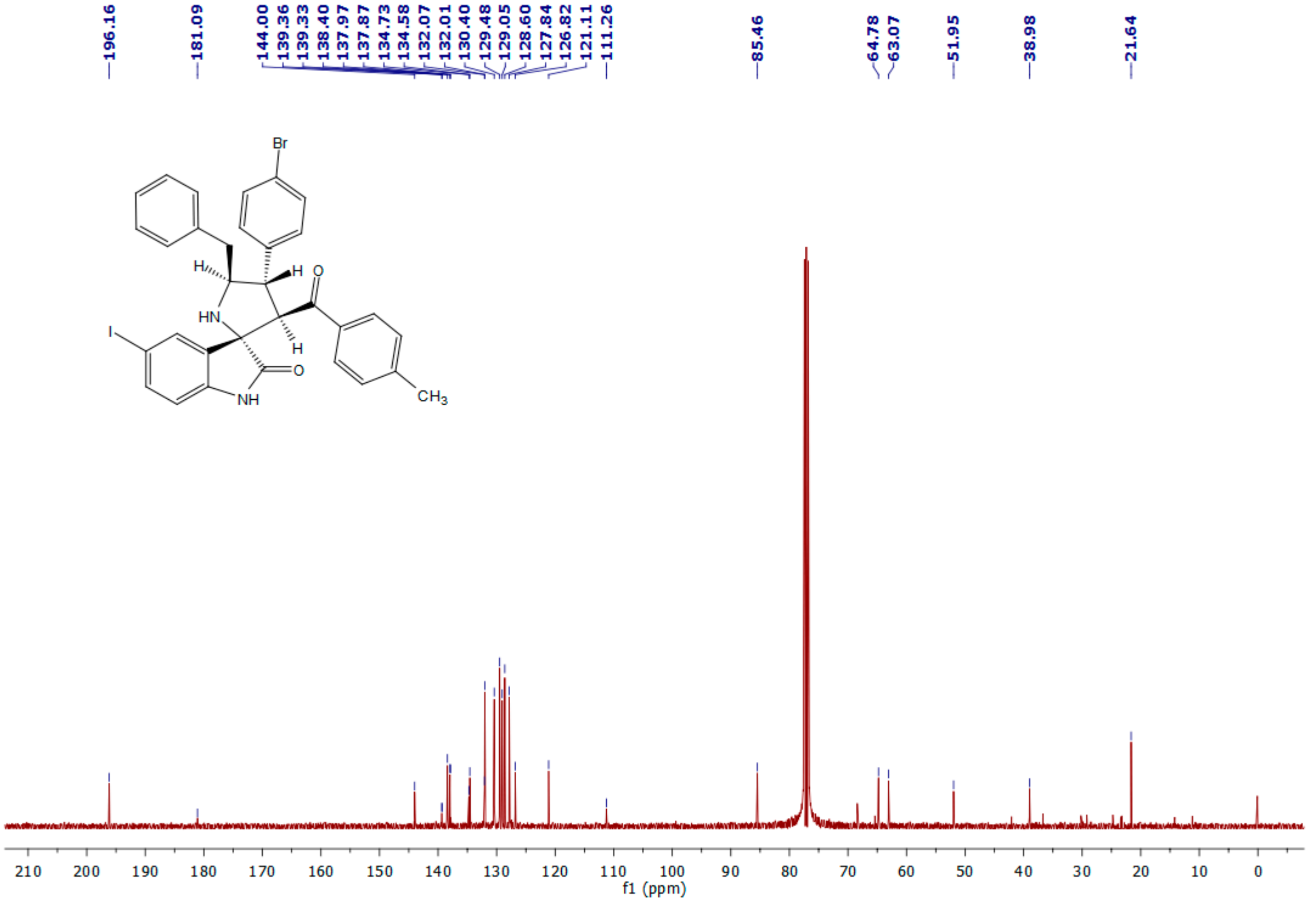

Figure S24: ^{13}C-NMR spectra of compound **24f**.

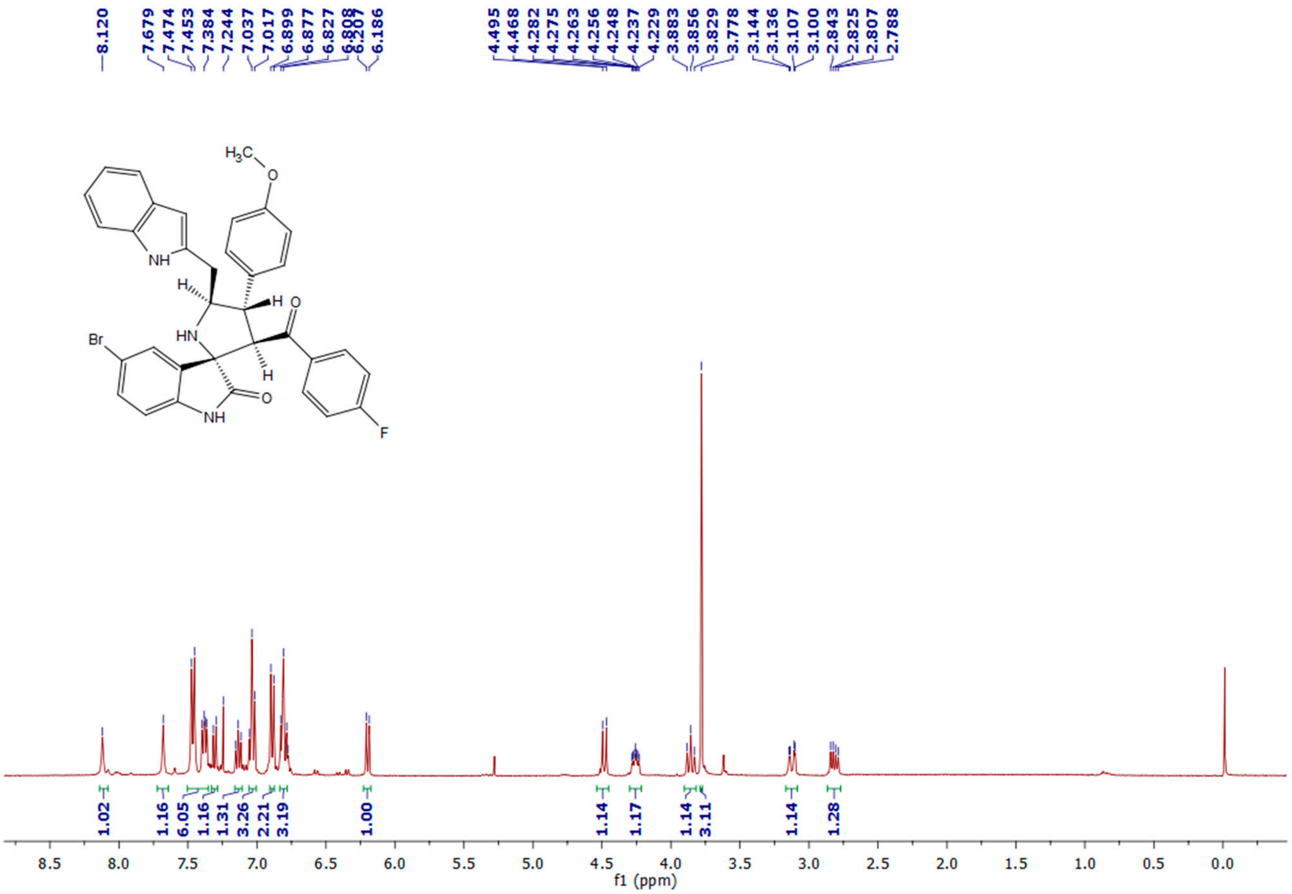

Figure S25: ^{1}H-NMR spectra of compound **25a**.

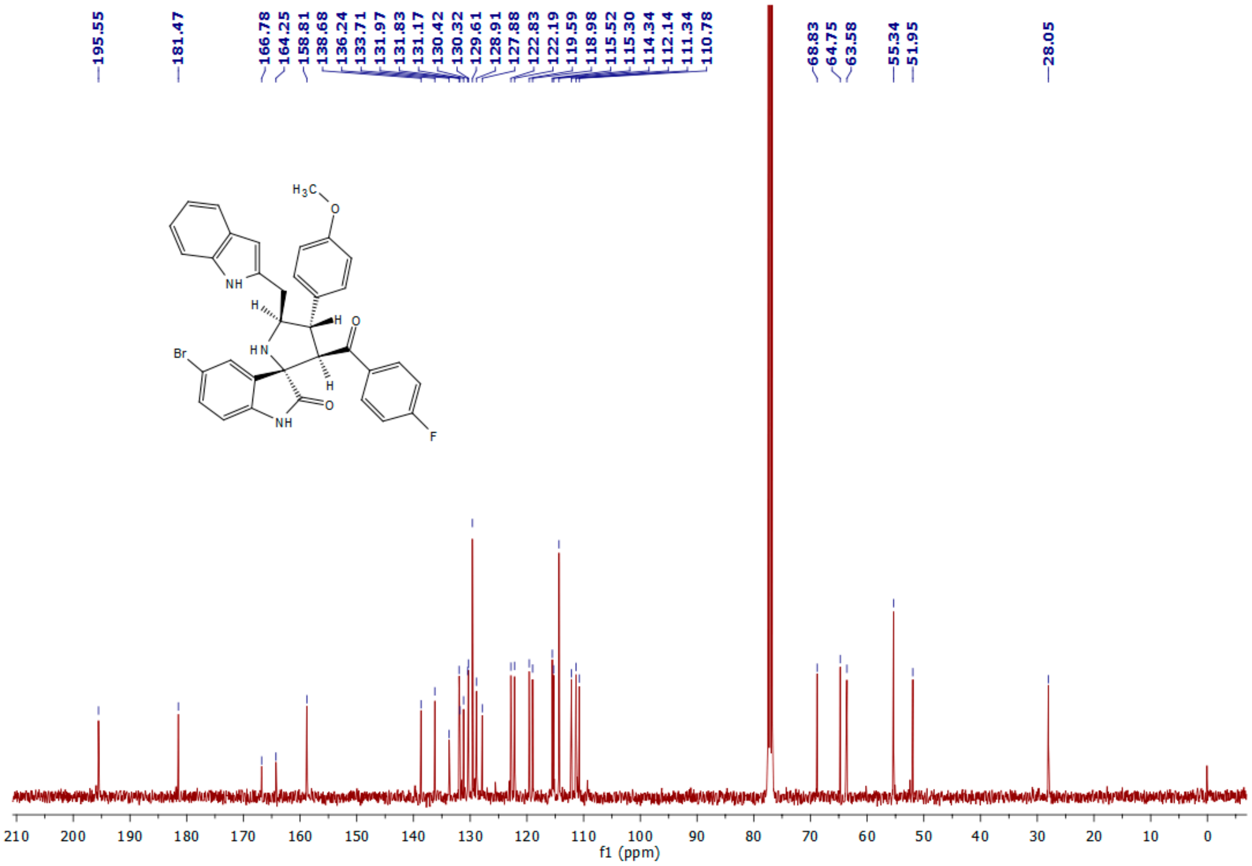

Figure S26: ^{13}C-NMR spectra of compound **25a**.

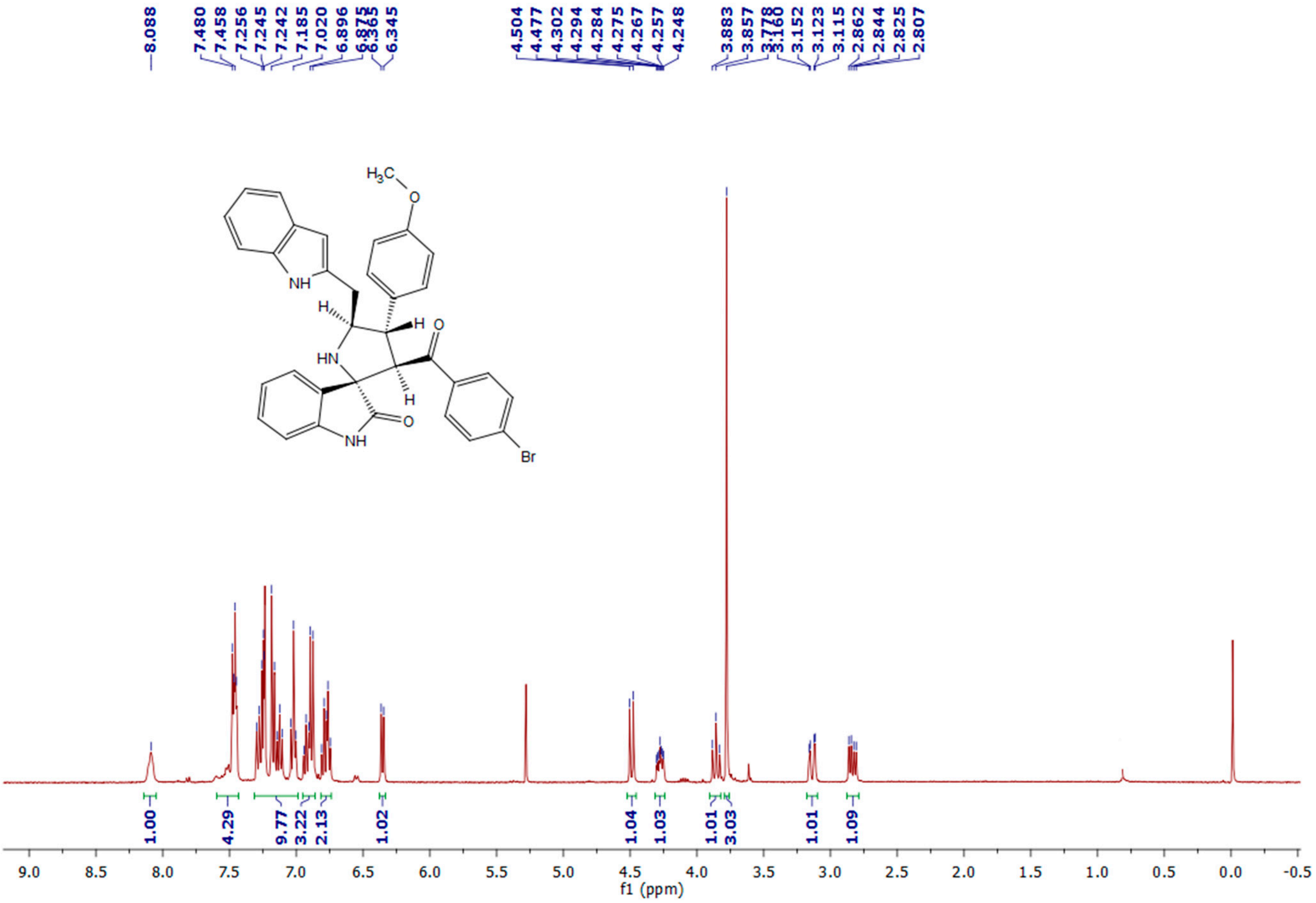

Figure S27: ^{1}H-NMR spectra of compound **25b**.

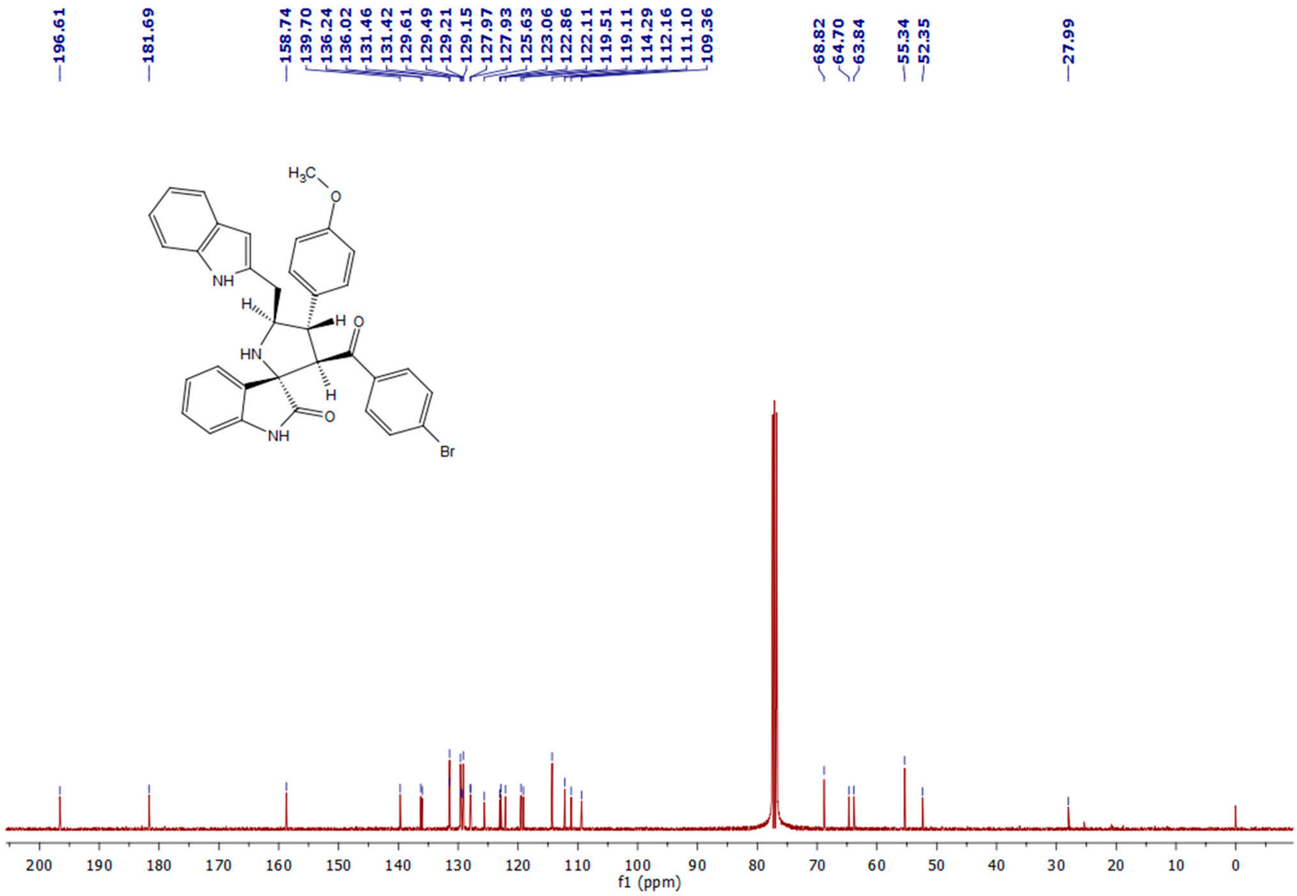

Figure S28: ^{13}C-NMR spectra of compound **25b**.

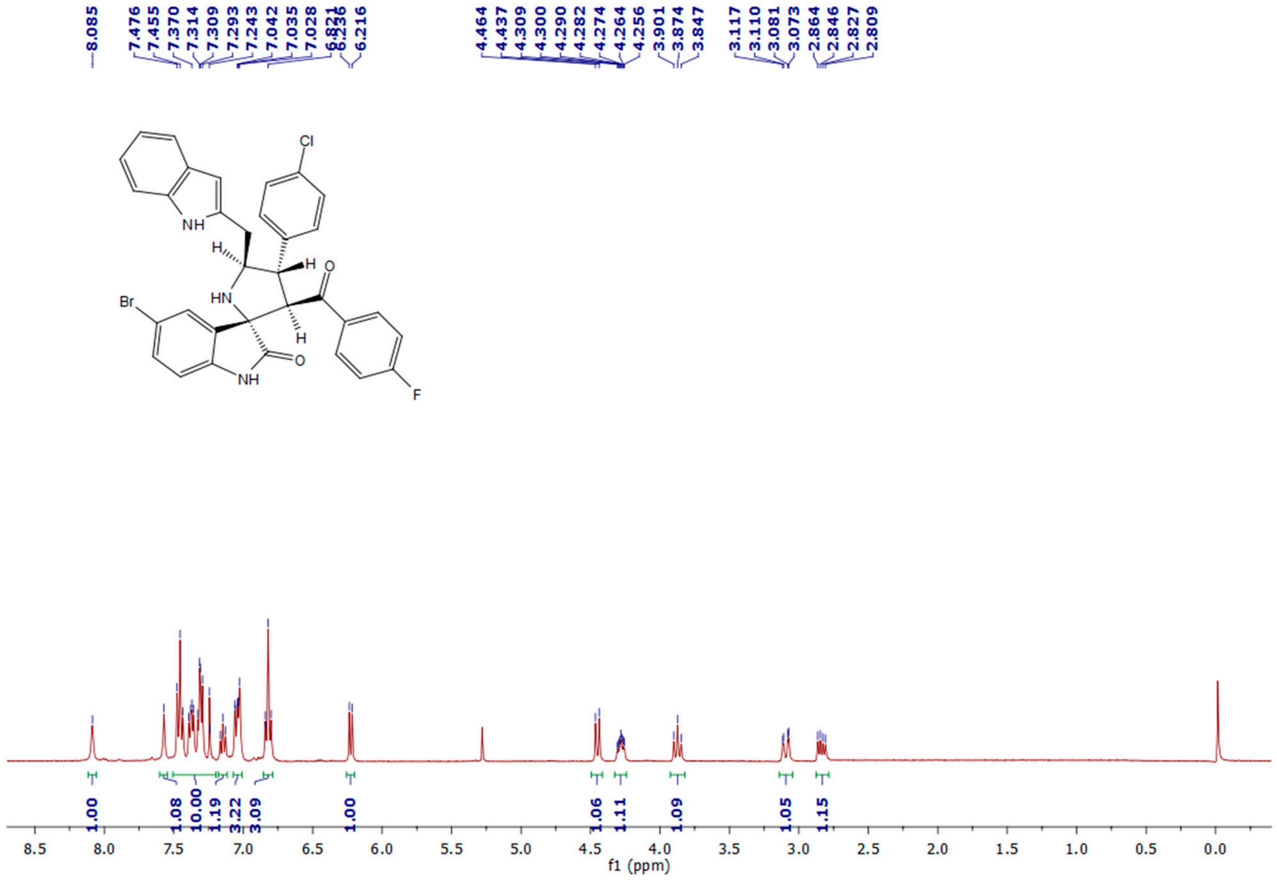

Figure S29: ^{1}H-NMR spectra of compound **25c**.

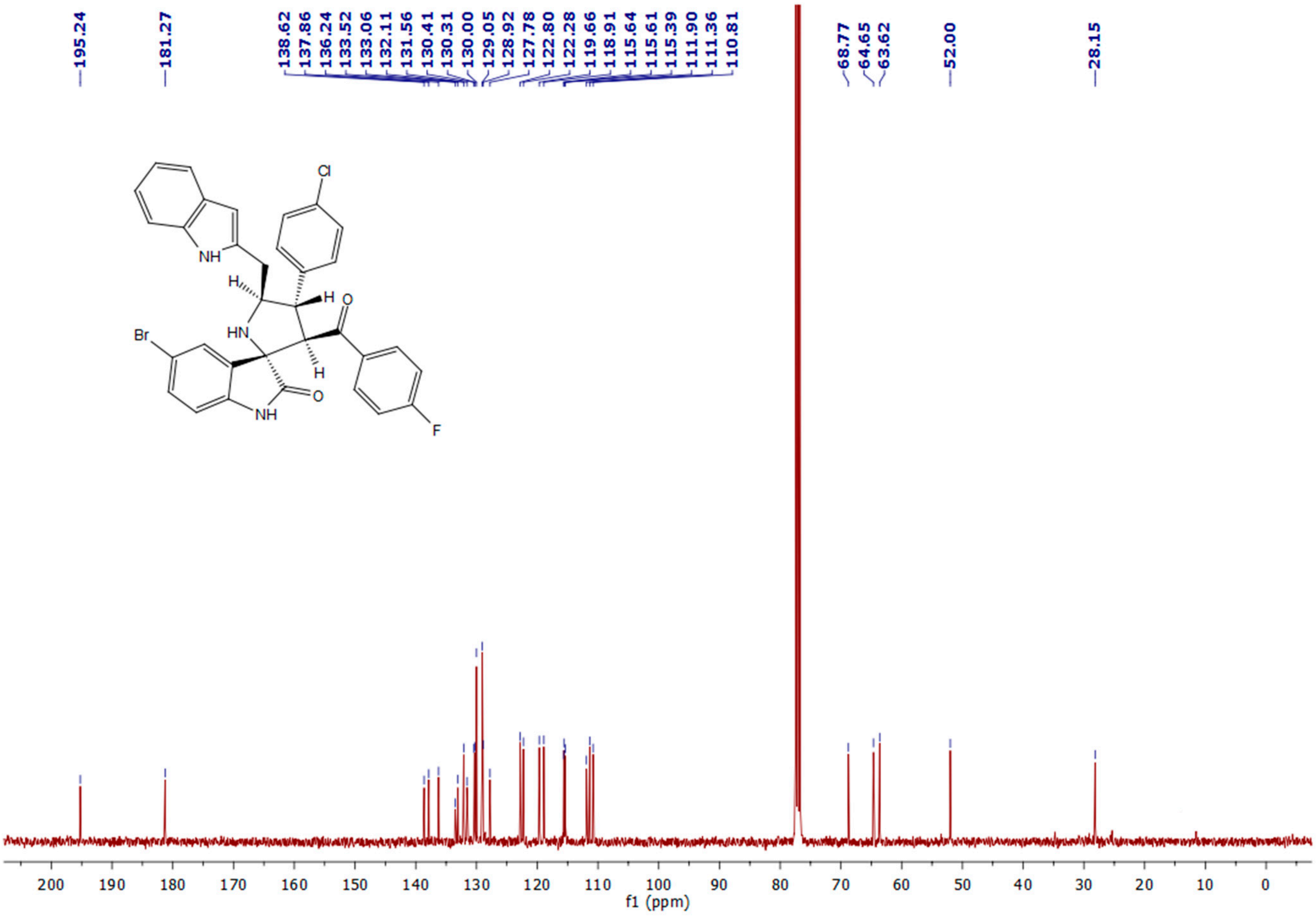

Figure S30: ^{13}C-NMR spectra of compound **25c**.

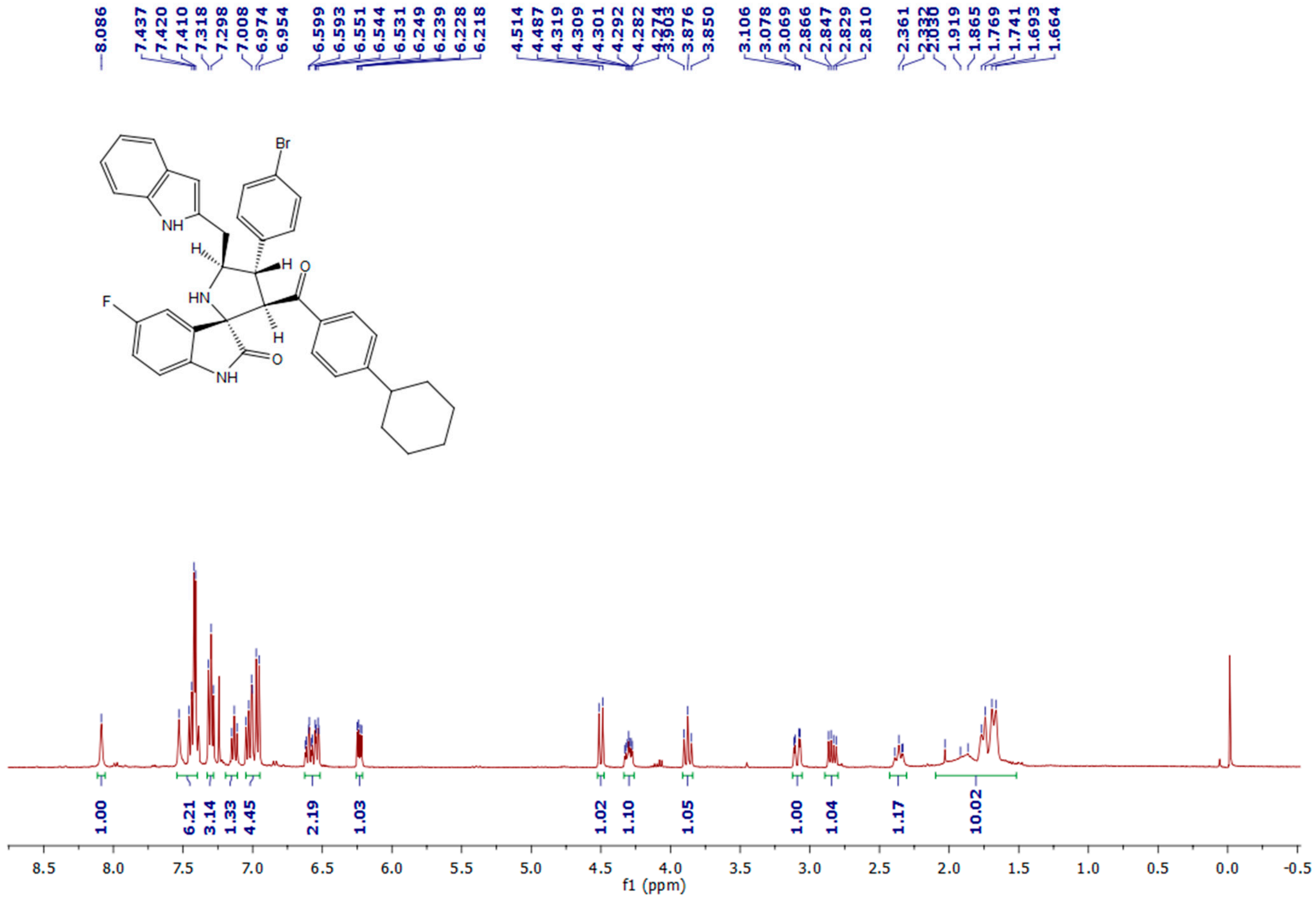

Figure S31: ^{1}H-NMR spectra of compound **25d**.

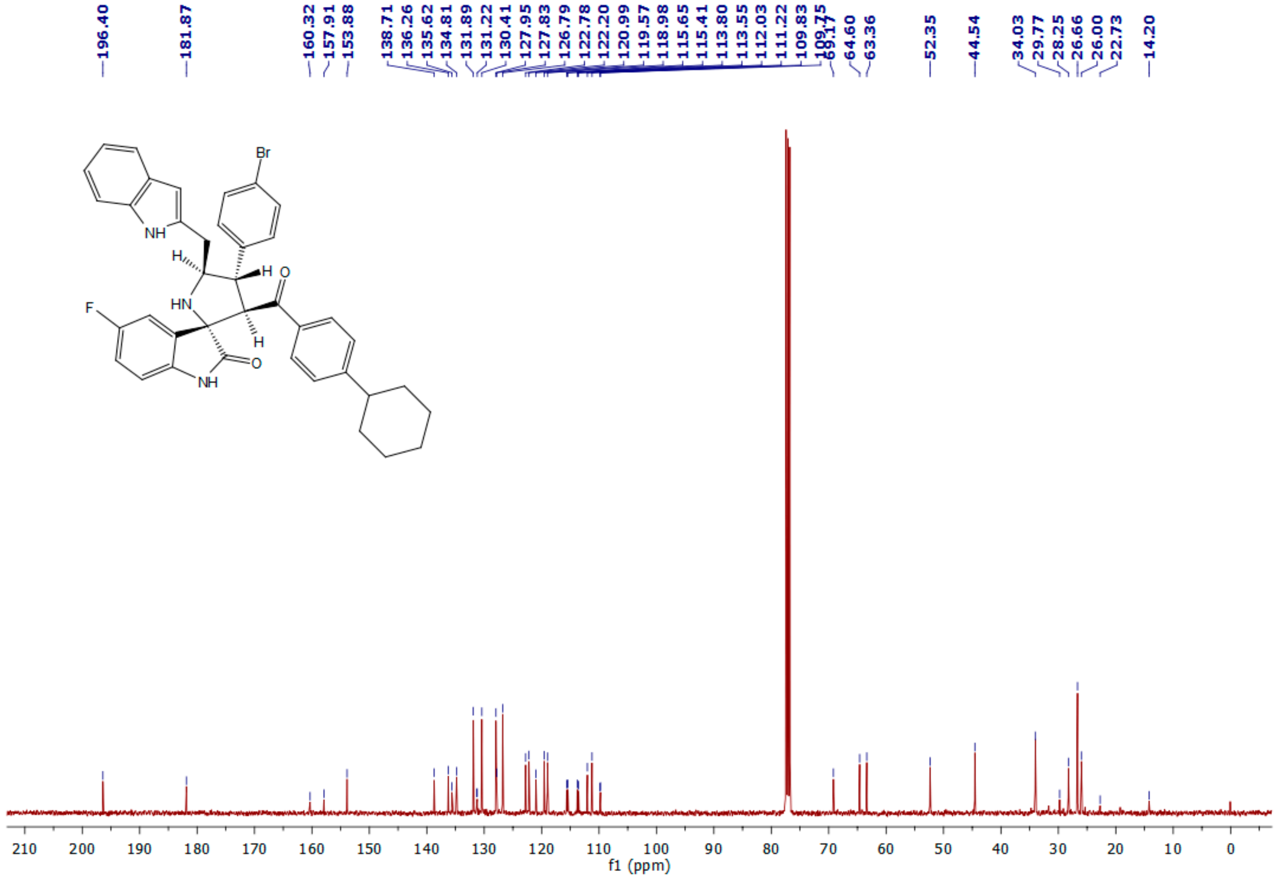

Figure S32: ^{13}C-NMR spectra of compound **25d**.

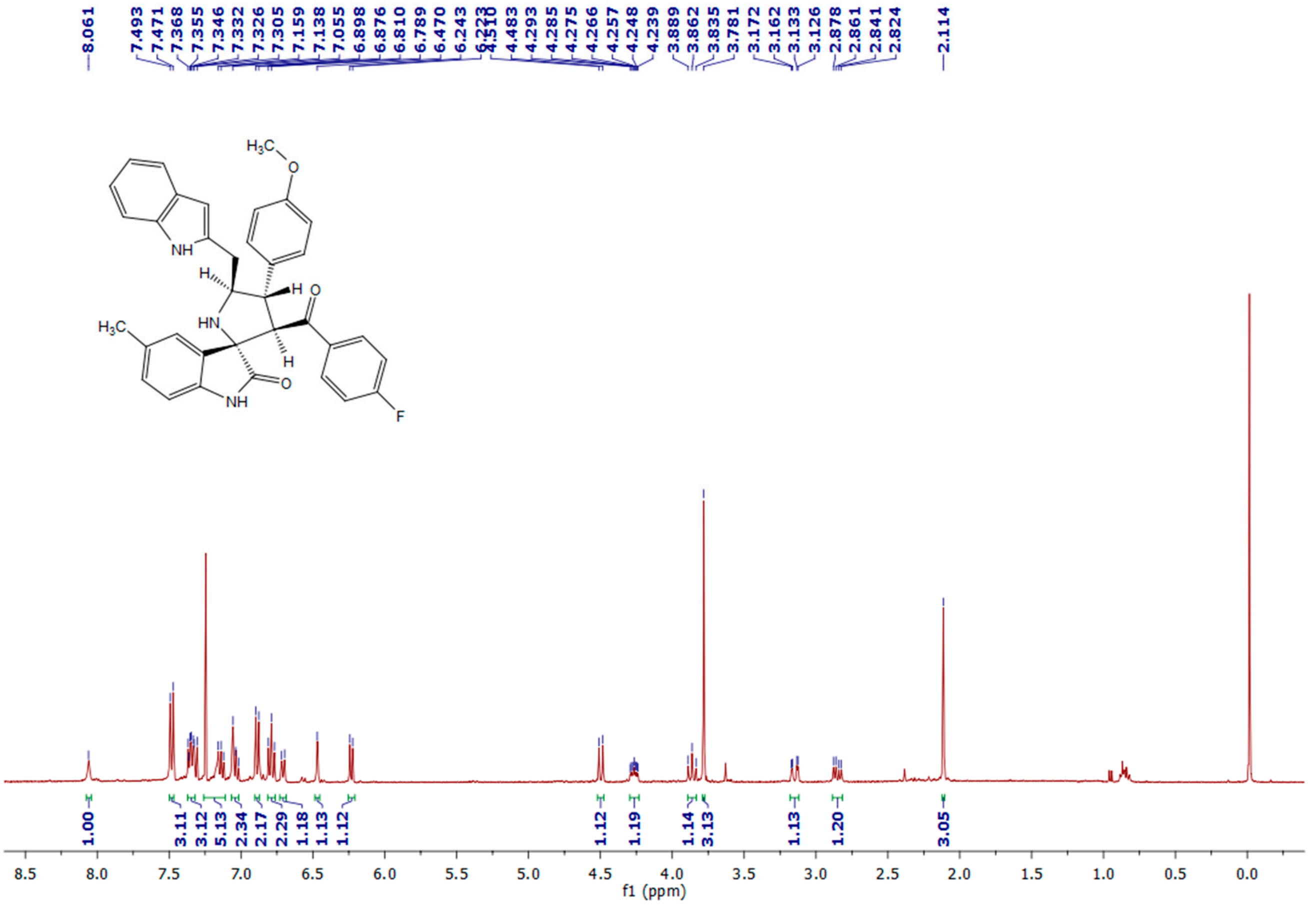

Figure S33: ^{1}H-NMR spectra of compound **25e**.

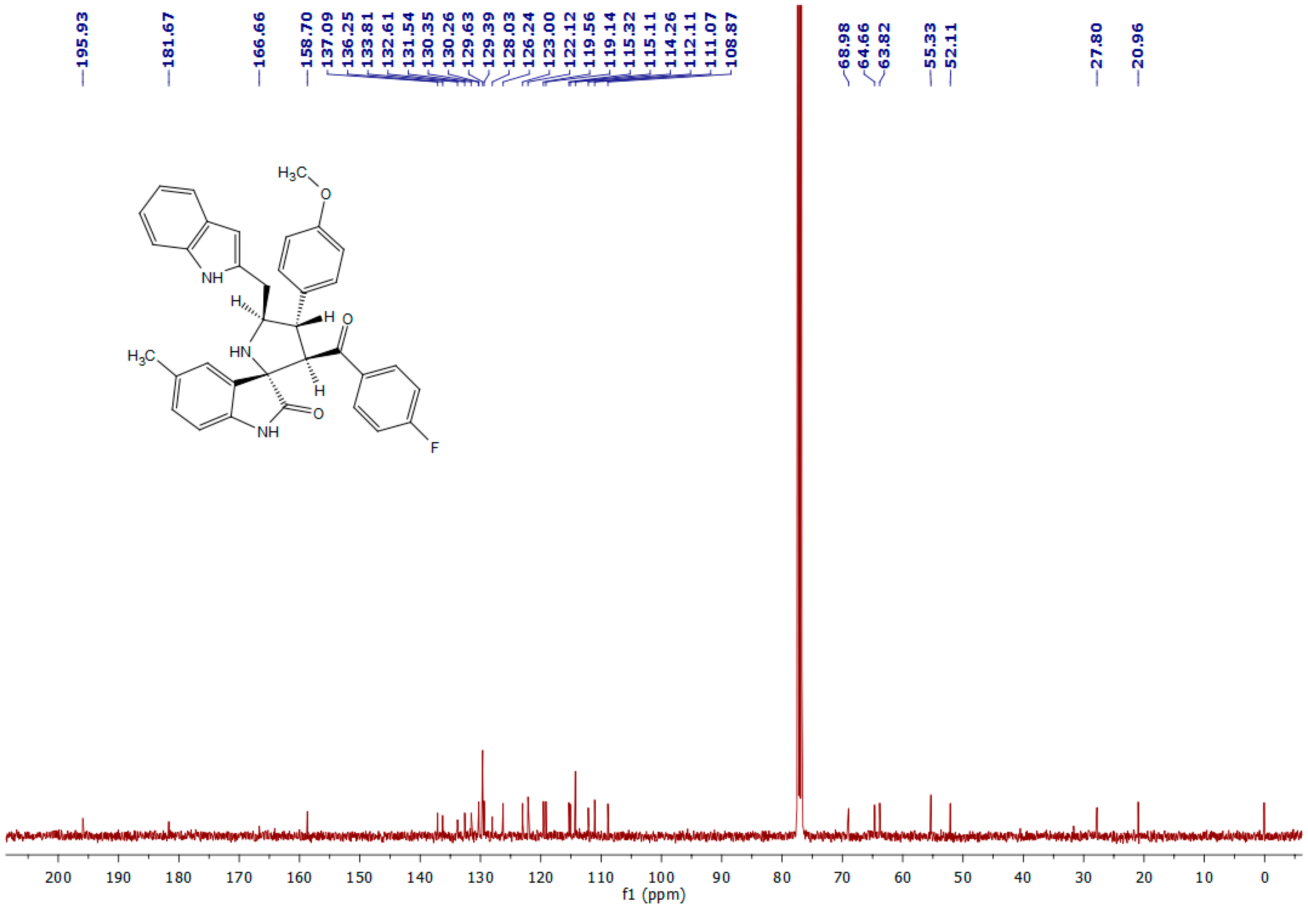

Figure S34: ^{13}C-NMR spectra of compound **25e**.

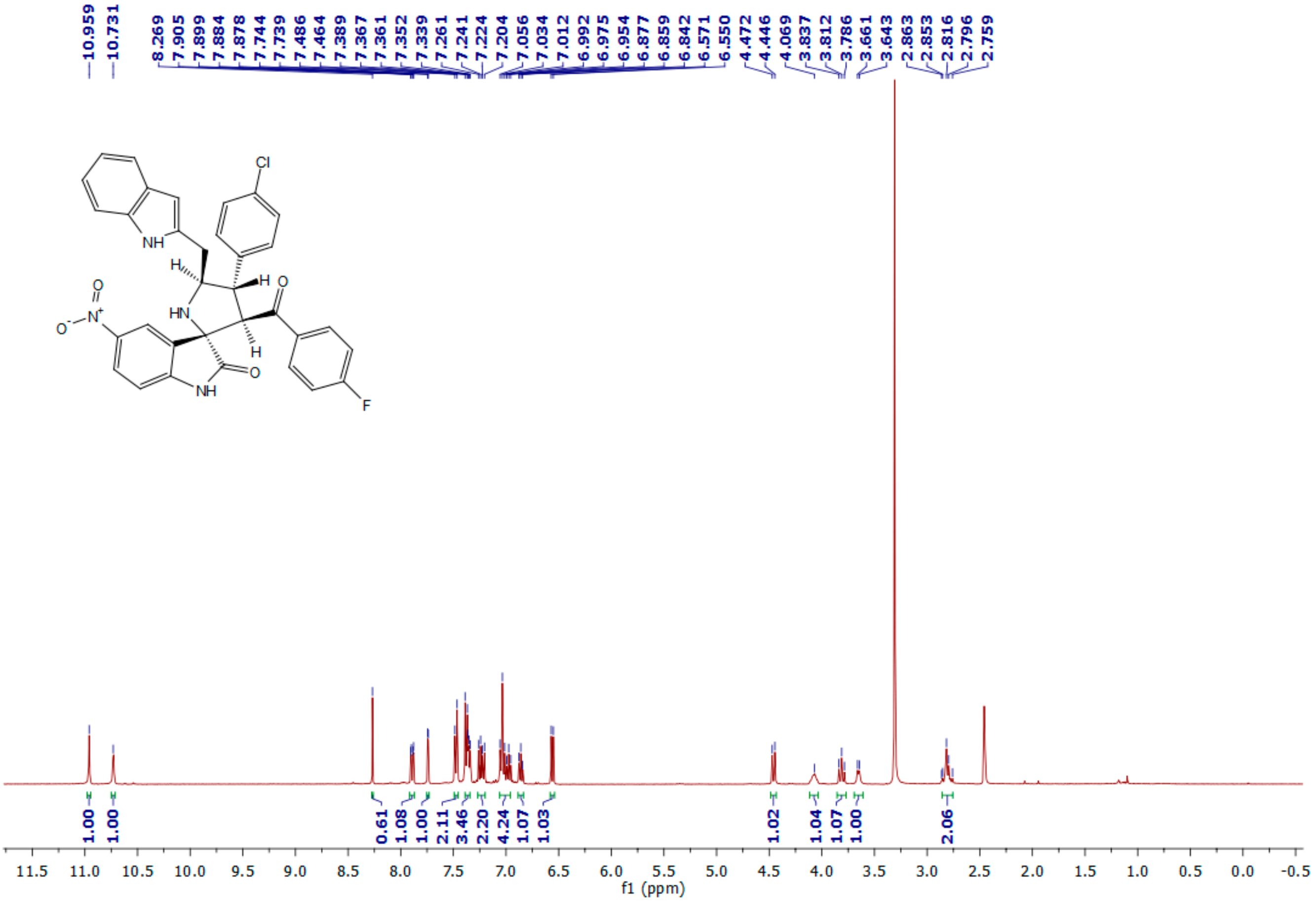

Figure S35: ^{1}H-NMR spectra of compound **25f**.

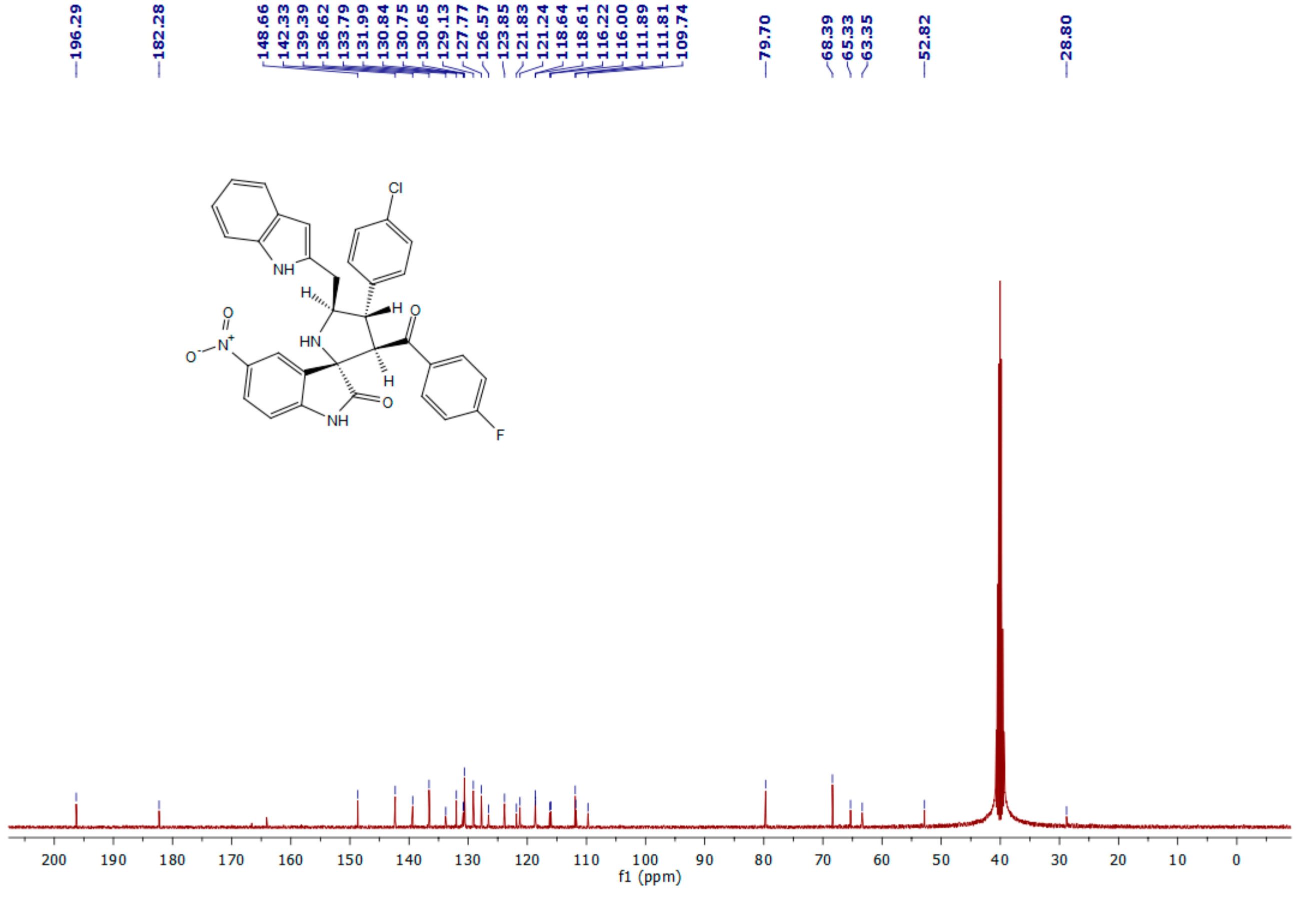

Figure S36: ^{13}C-NMR spectra of compound **25f**.

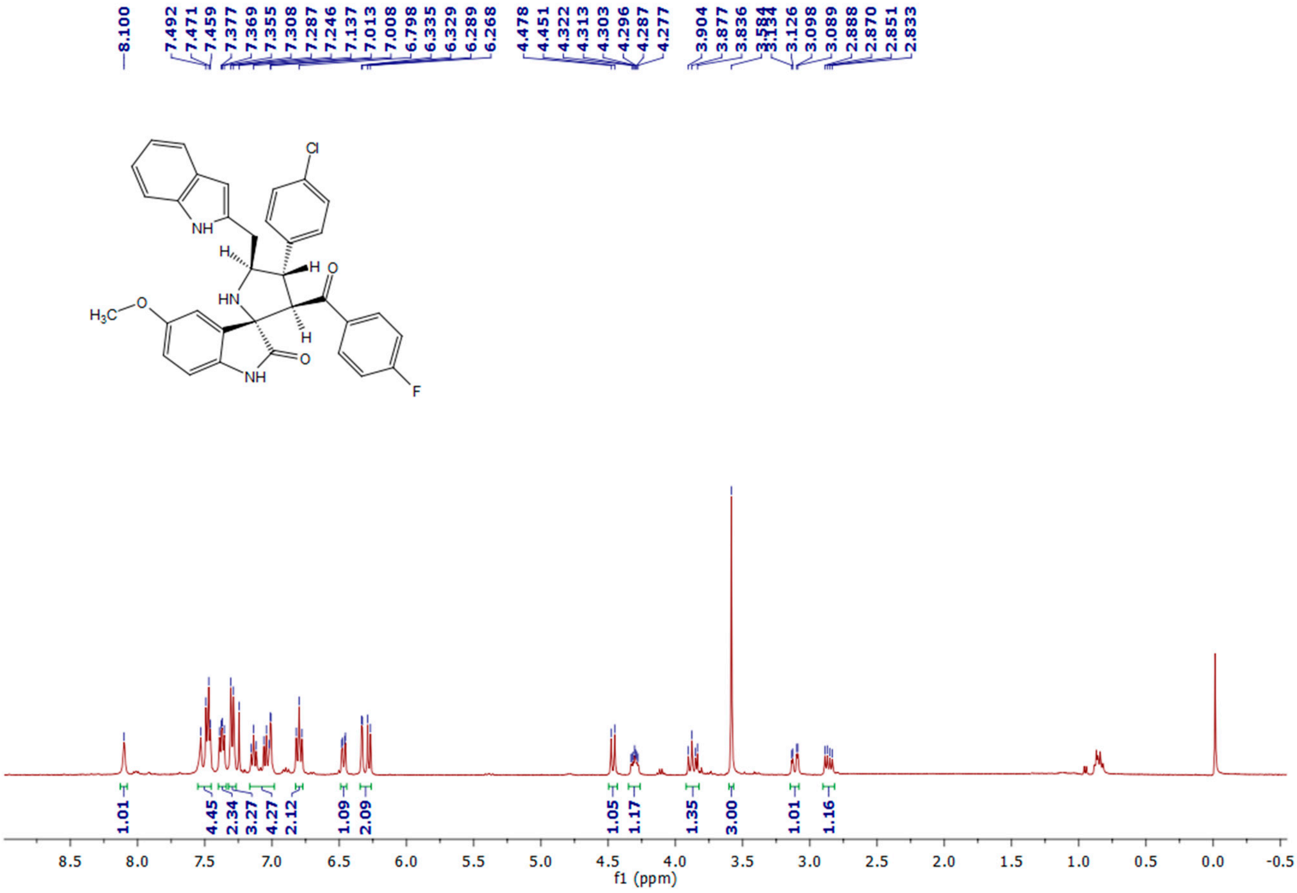

Figure S37: ^{1}H-NMR spectra of compound **25g**.

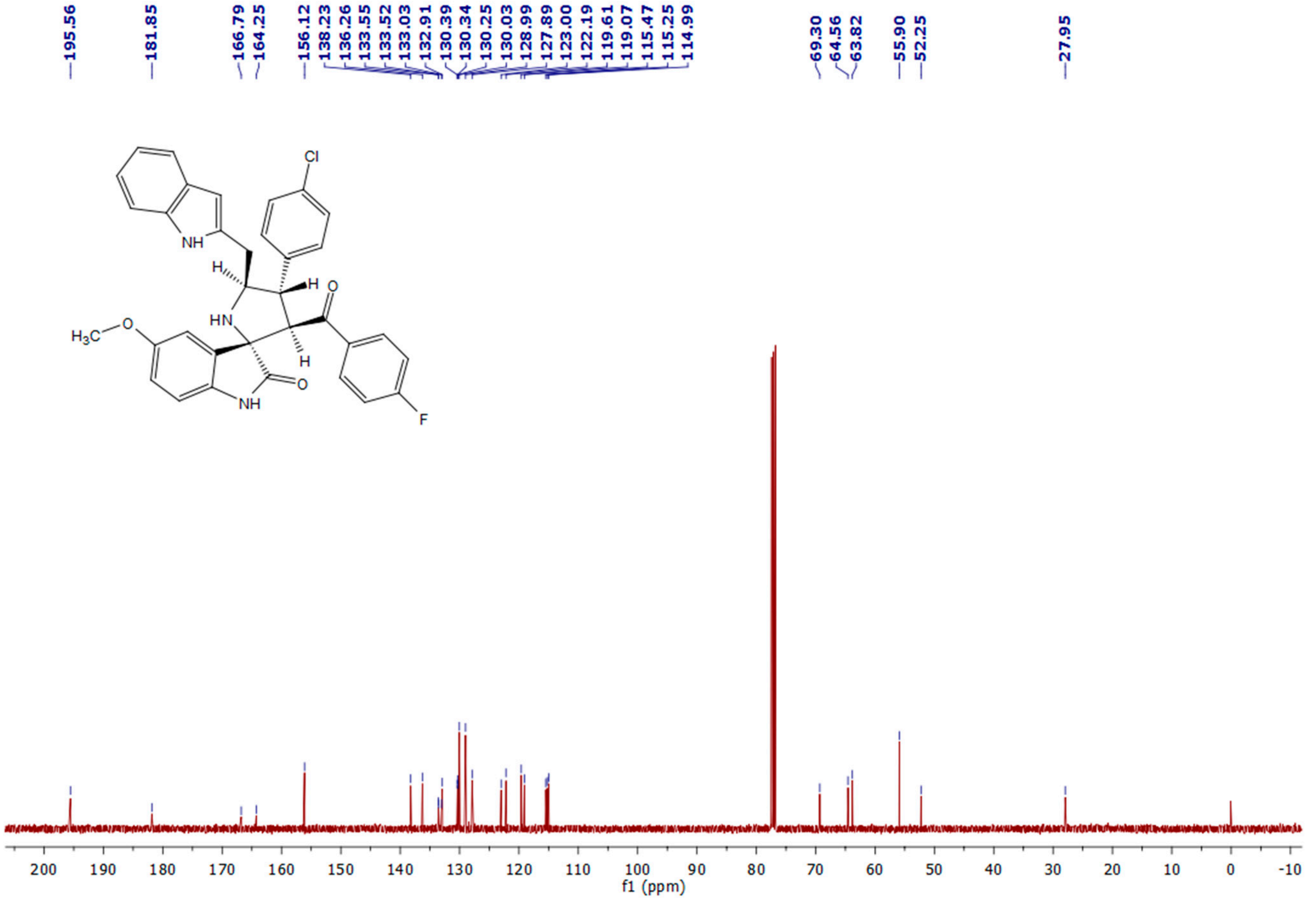

Figure S38: ^{13}C-NMR spectra of compound **25g**.
